# Supplementary figures and images for: Spatiotemporal prediction of alpine wetlands under multi-climate scenarios in the west of Sichuan, China
Source: PeerJ. 2024 Nov 27;12:e18586. doi: 10.7717/peerj.18586 (PMC11608022; doi:10.7717/peerj.18586)

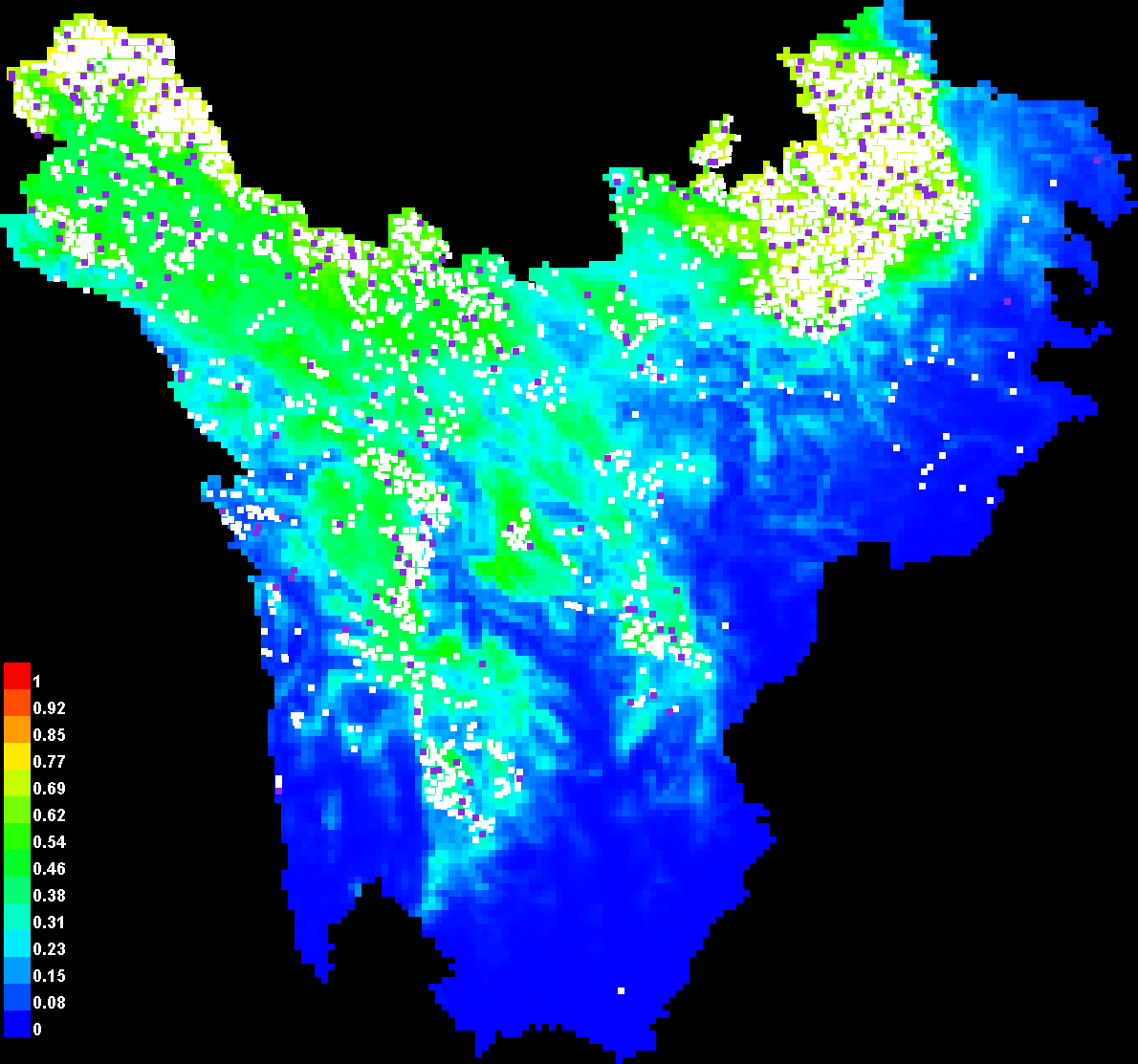

Supplement: Supplemental Information 1 [file peerj-12-18586-s001.zip › Maxent_data1/2.5mBIOssp126_2021-2040/RES/plots/wetland_0.png]

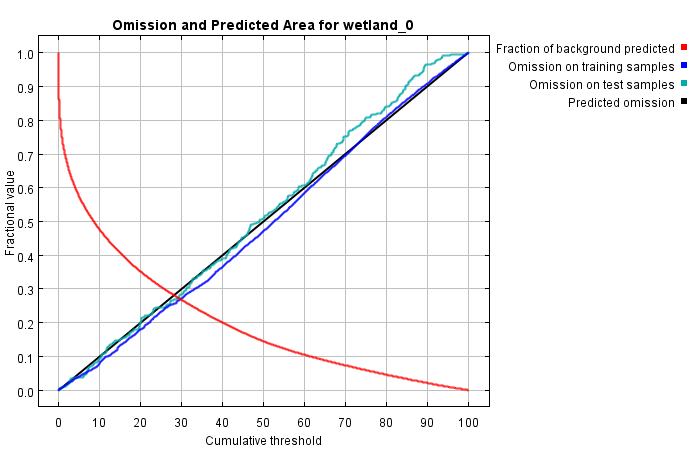

Supplement: Supplemental Information 1 [file peerj-12-18586-s001.zip › Maxent_data1/2.5mBIOssp126_2021-2040/RES/plots/wetland_0_omission.png]

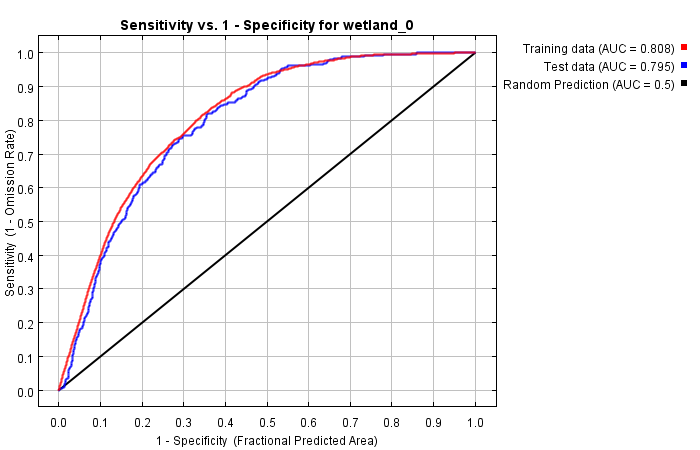

Supplement: Supplemental Information 1 [file peerj-12-18586-s001.zip › Maxent_data1/2.5mBIOssp126_2021-2040/RES/plots/wetland_0_roc.png]

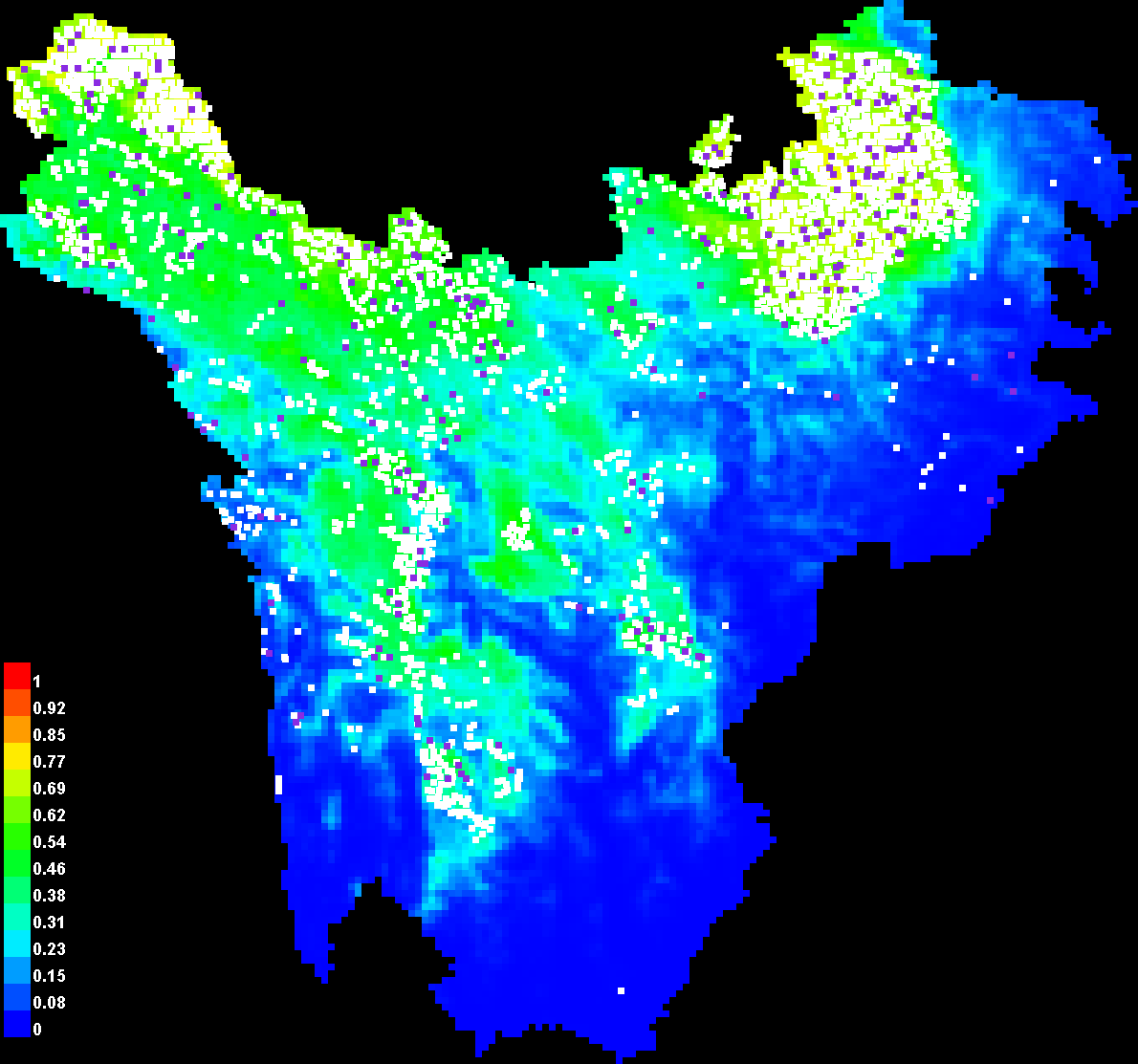

Supplement: Supplemental Information 1 [file peerj-12-18586-s001.zip › Maxent_data1/2.5mBIOssp126_2021-2040/RES/plots/wetland_1.png]

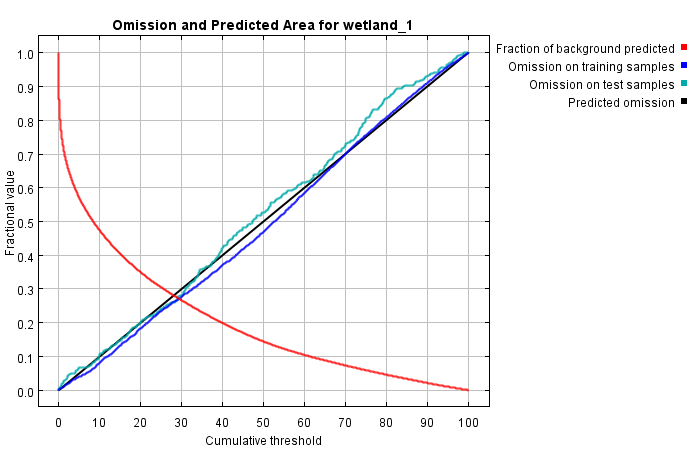

Supplement: Supplemental Information 1 [file peerj-12-18586-s001.zip › Maxent_data1/2.5mBIOssp126_2021-2040/RES/plots/wetland_1_omission.png]

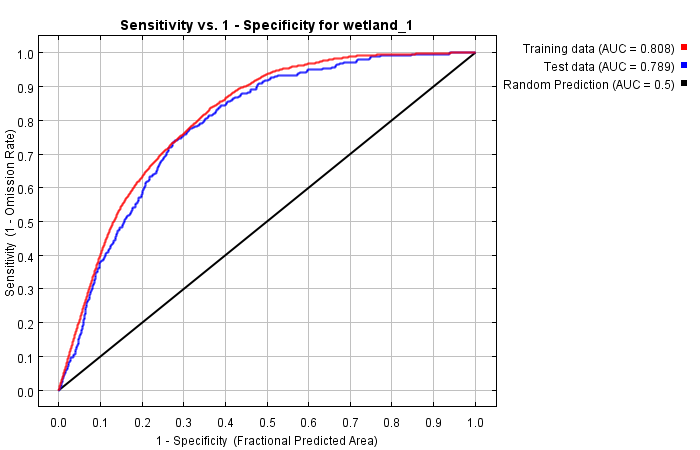

Supplement: Supplemental Information 1 [file peerj-12-18586-s001.zip › Maxent_data1/2.5mBIOssp126_2021-2040/RES/plots/wetland_1_roc.png]

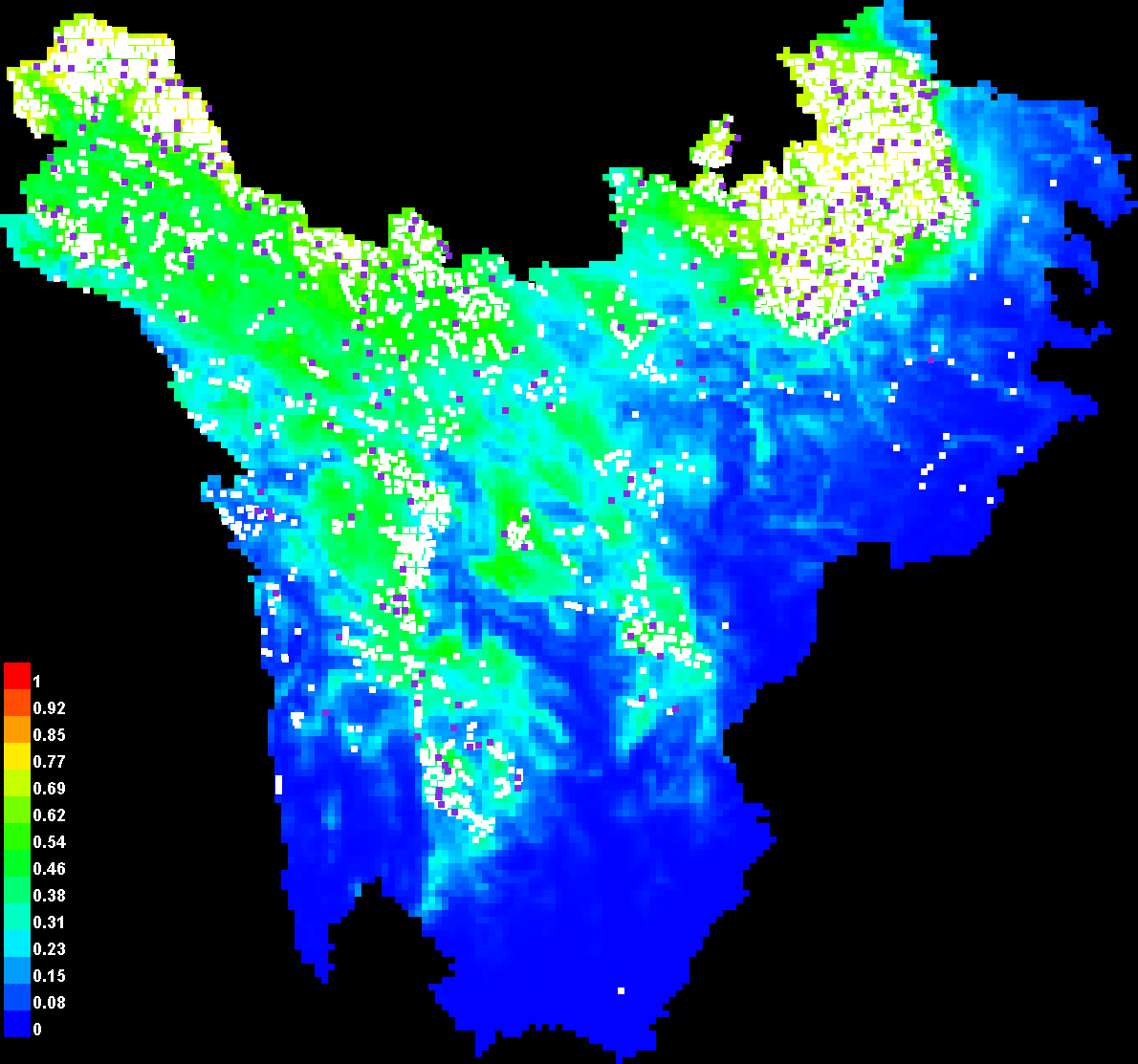

Supplement: Supplemental Information 1 [file peerj-12-18586-s001.zip › Maxent_data1/2.5mBIOssp126_2021-2040/RES/plots/wetland_2.png]

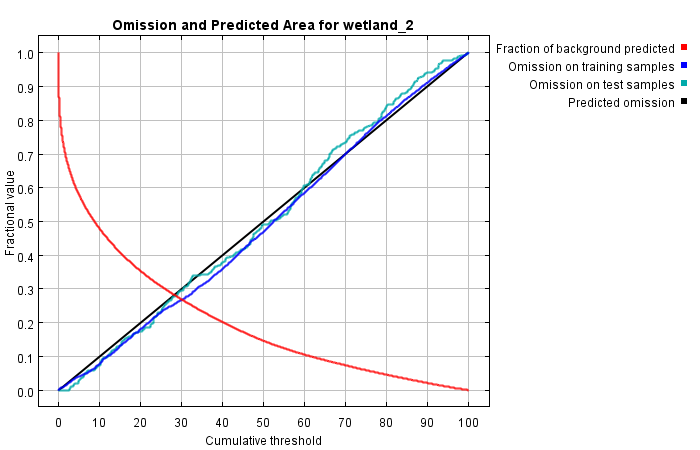

Supplement: Supplemental Information 1 [file peerj-12-18586-s001.zip › Maxent_data1/2.5mBIOssp126_2021-2040/RES/plots/wetland_2_omission.png]

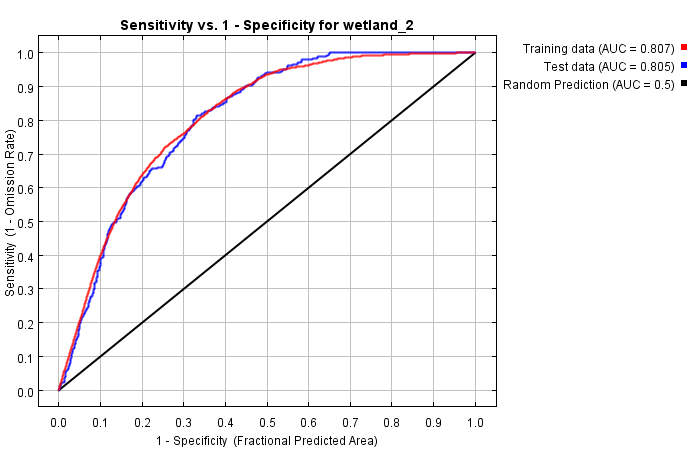

Supplement: Supplemental Information 1 [file peerj-12-18586-s001.zip › Maxent_data1/2.5mBIOssp126_2021-2040/RES/plots/wetland_2_roc.png]

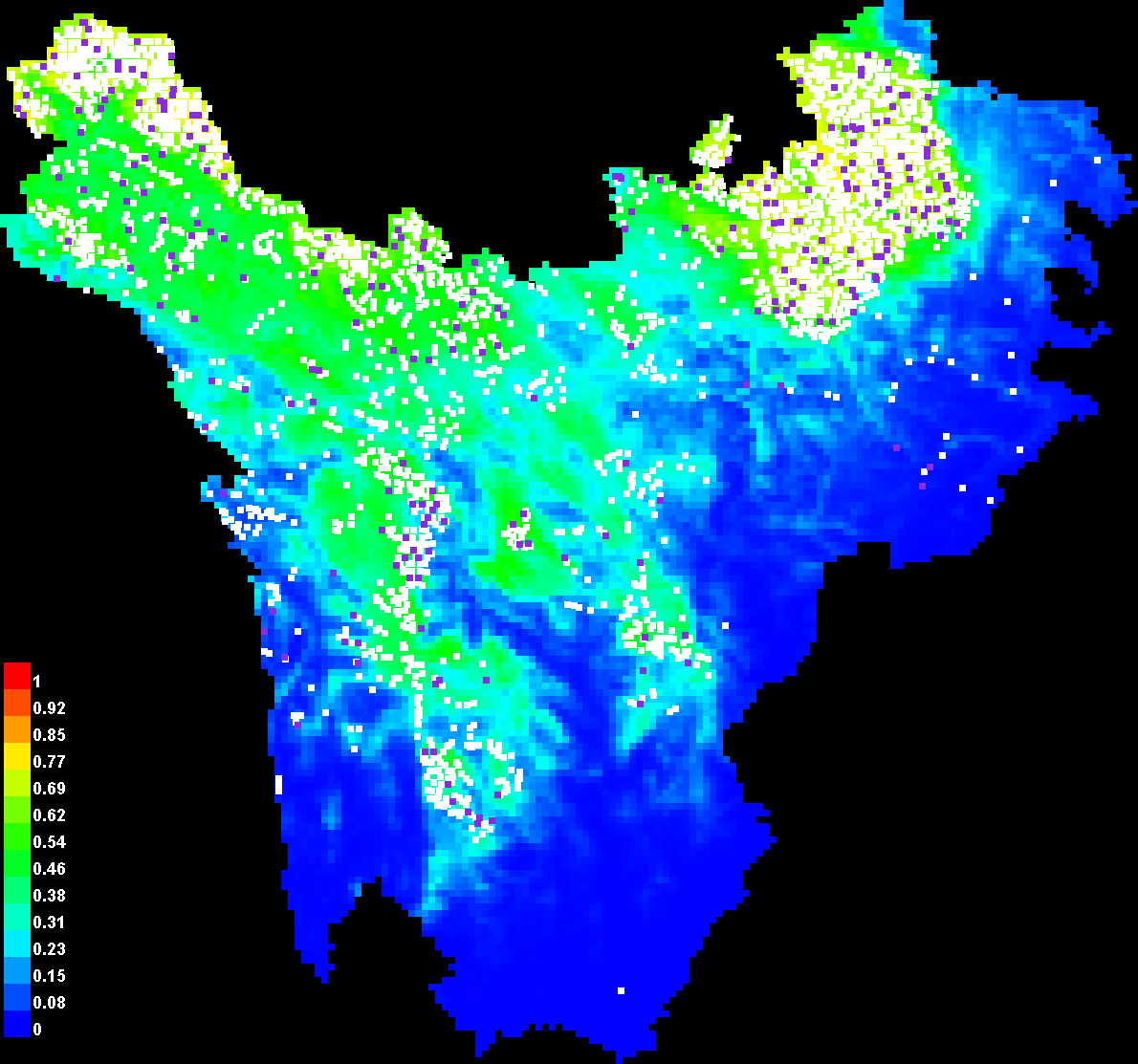

Supplement: Supplemental Information 1 [file peerj-12-18586-s001.zip › Maxent_data1/2.5mBIOssp126_2021-2040/RES/plots/wetland_3.png]

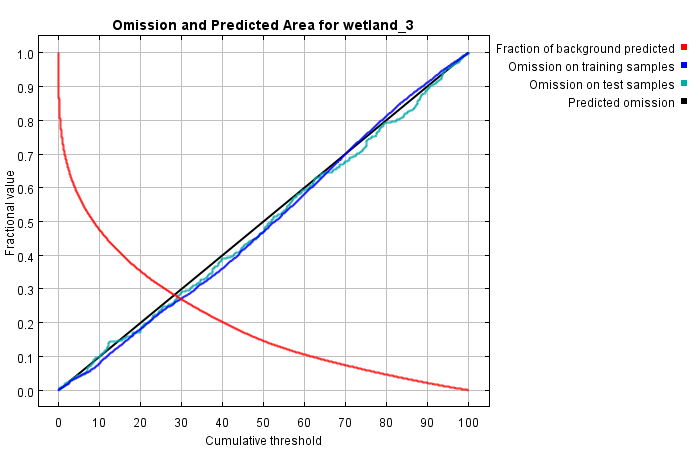

Supplement: Supplemental Information 1 [file peerj-12-18586-s001.zip › Maxent_data1/2.5mBIOssp126_2021-2040/RES/plots/wetland_3_omission.png]

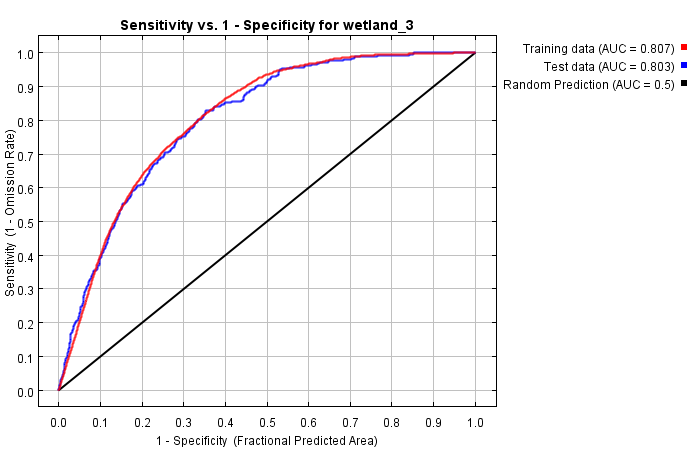

Supplement: Supplemental Information 1 [file peerj-12-18586-s001.zip › Maxent_data1/2.5mBIOssp126_2021-2040/RES/plots/wetland_3_roc.png]

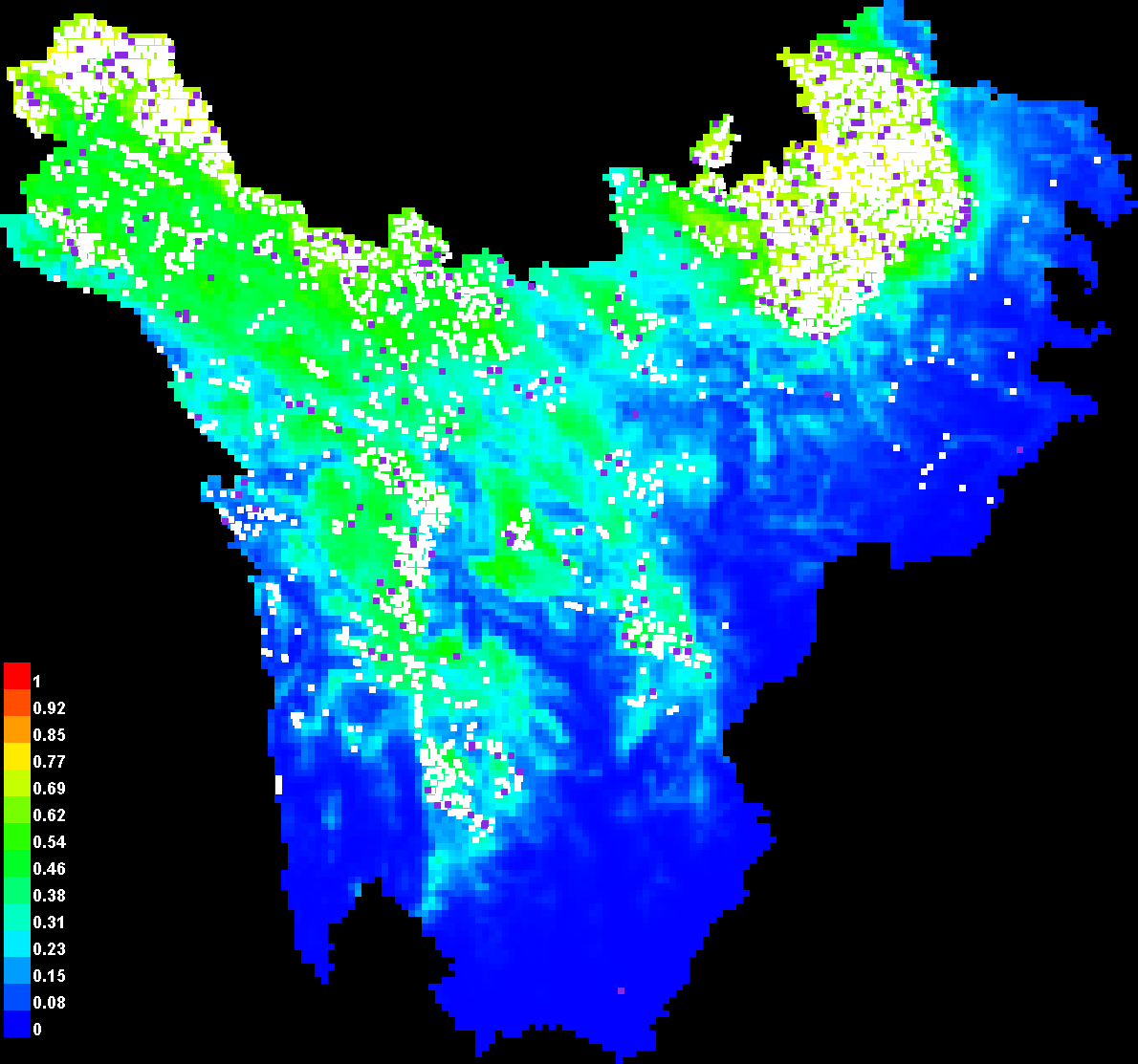

Supplement: Supplemental Information 1 [file peerj-12-18586-s001.zip › Maxent_data1/2.5mBIOssp126_2021-2040/RES/plots/wetland_4.png]

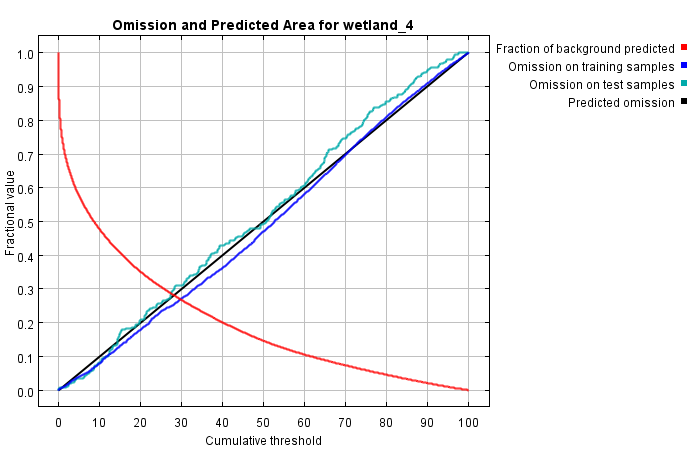

Supplement: Supplemental Information 1 [file peerj-12-18586-s001.zip › Maxent_data1/2.5mBIOssp126_2021-2040/RES/plots/wetland_4_omission.png]

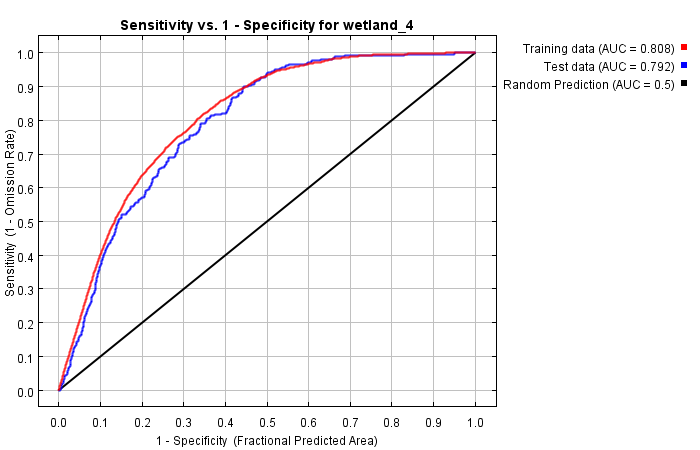

Supplement: Supplemental Information 1 [file peerj-12-18586-s001.zip › Maxent_data1/2.5mBIOssp126_2021-2040/RES/plots/wetland_4_roc.png]

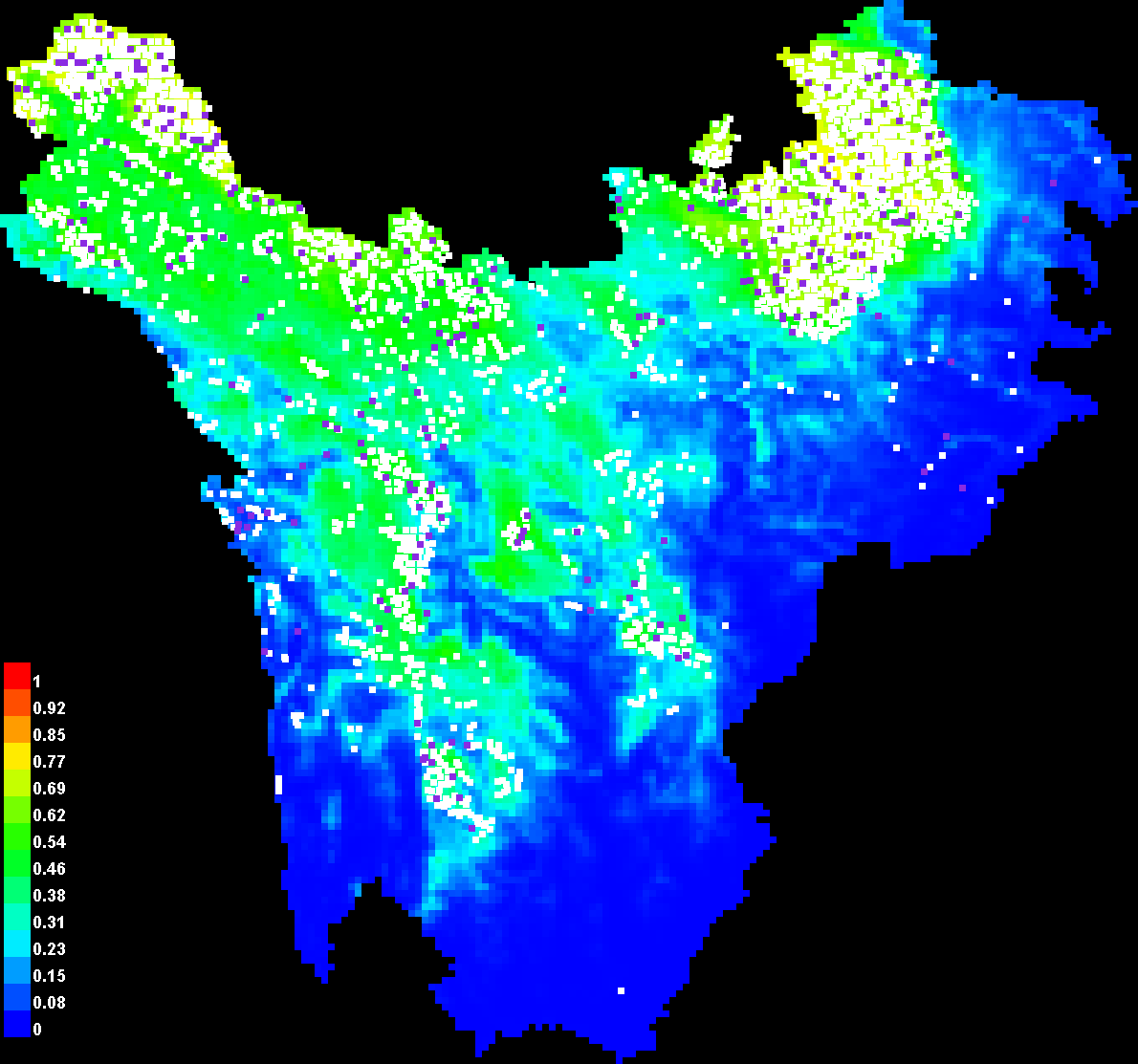

Supplement: Supplemental Information 1 [file peerj-12-18586-s001.zip › Maxent_data1/2.5mBIOssp126_2021-2040/RES/plots/wetland_5.png]

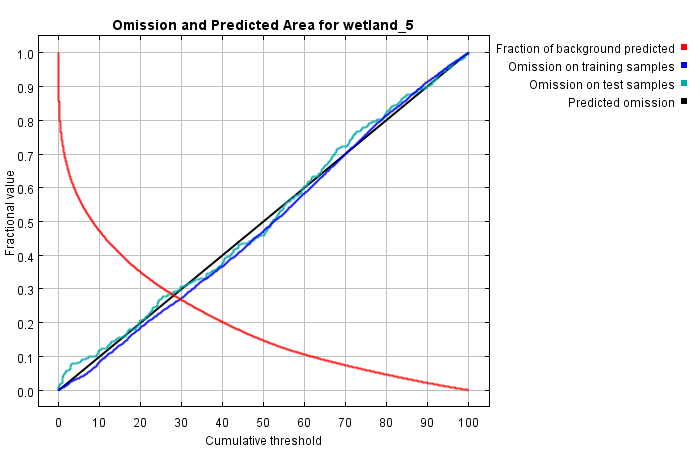

Supplement: Supplemental Information 1 [file peerj-12-18586-s001.zip › Maxent_data1/2.5mBIOssp126_2021-2040/RES/plots/wetland_5_omission.png]

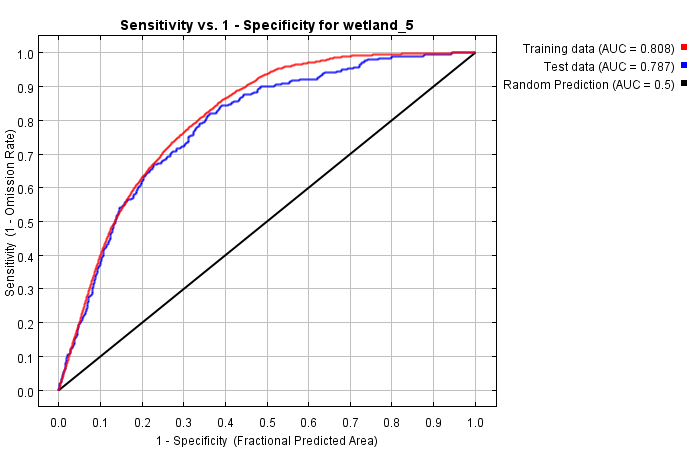

Supplement: Supplemental Information 1 [file peerj-12-18586-s001.zip › Maxent_data1/2.5mBIOssp126_2021-2040/RES/plots/wetland_5_roc.png]

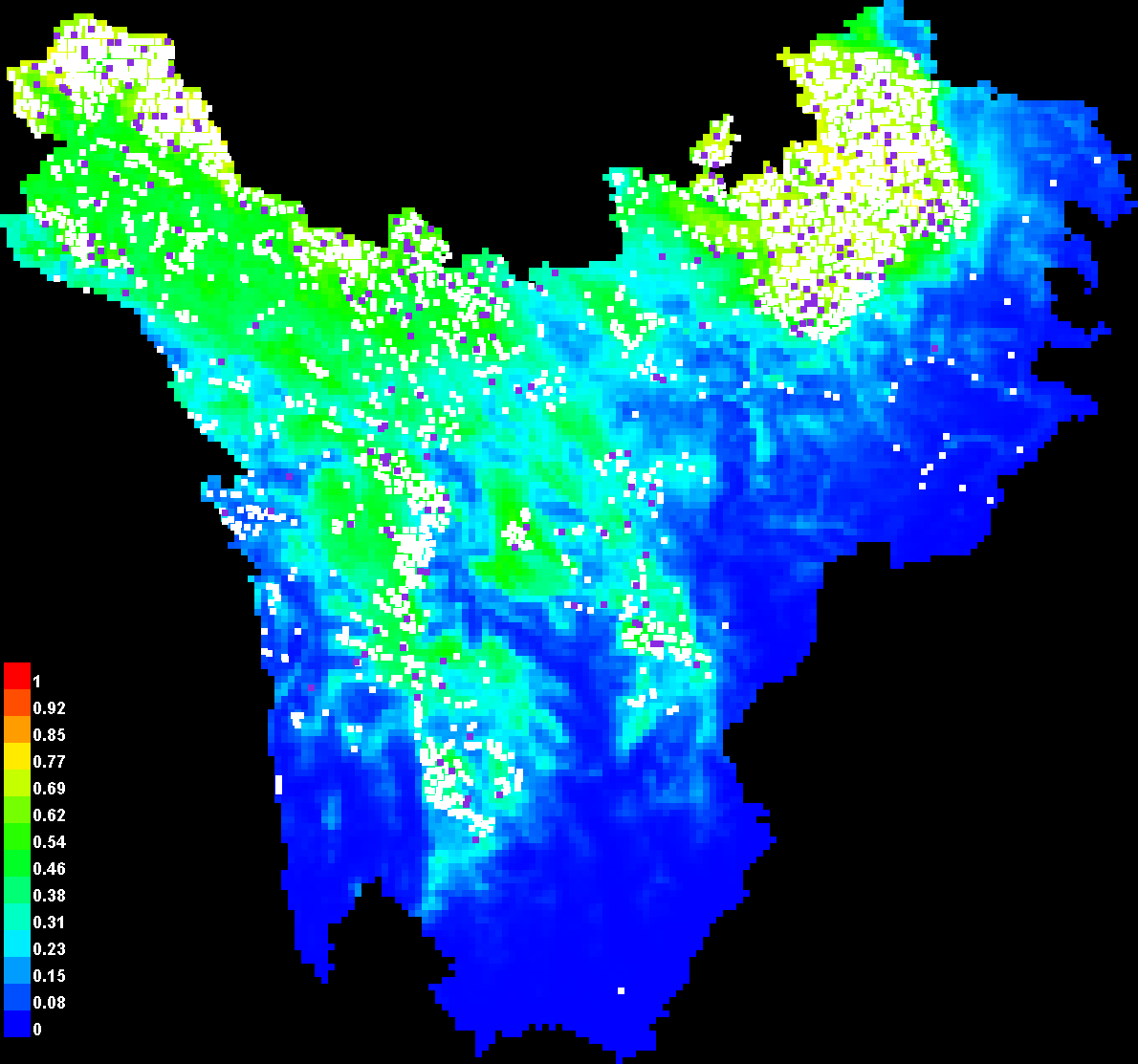

Supplement: Supplemental Information 1 [file peerj-12-18586-s001.zip › Maxent_data1/2.5mBIOssp126_2021-2040/RES/plots/wetland_6.png]

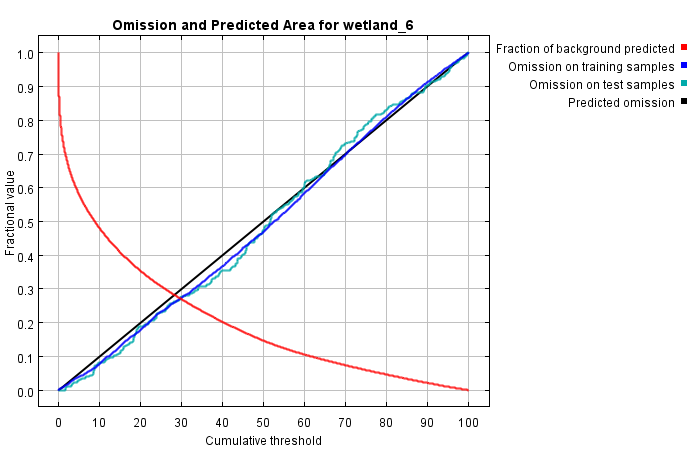

Supplement: Supplemental Information 1 [file peerj-12-18586-s001.zip › Maxent_data1/2.5mBIOssp126_2021-2040/RES/plots/wetland_6_omission.png]

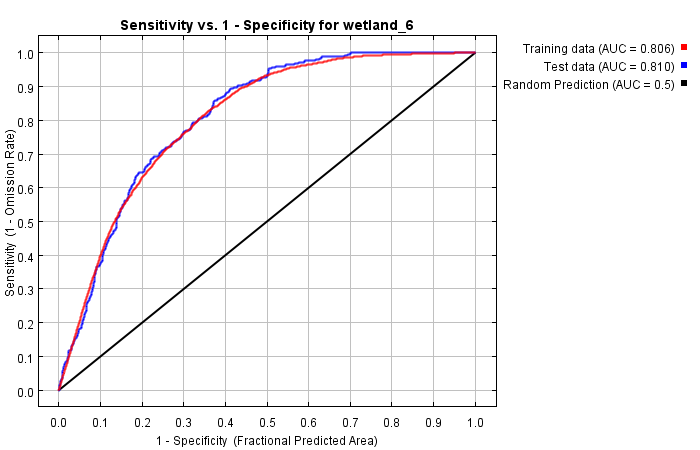

Supplement: Supplemental Information 1 [file peerj-12-18586-s001.zip › Maxent_data1/2.5mBIOssp126_2021-2040/RES/plots/wetland_6_roc.png]

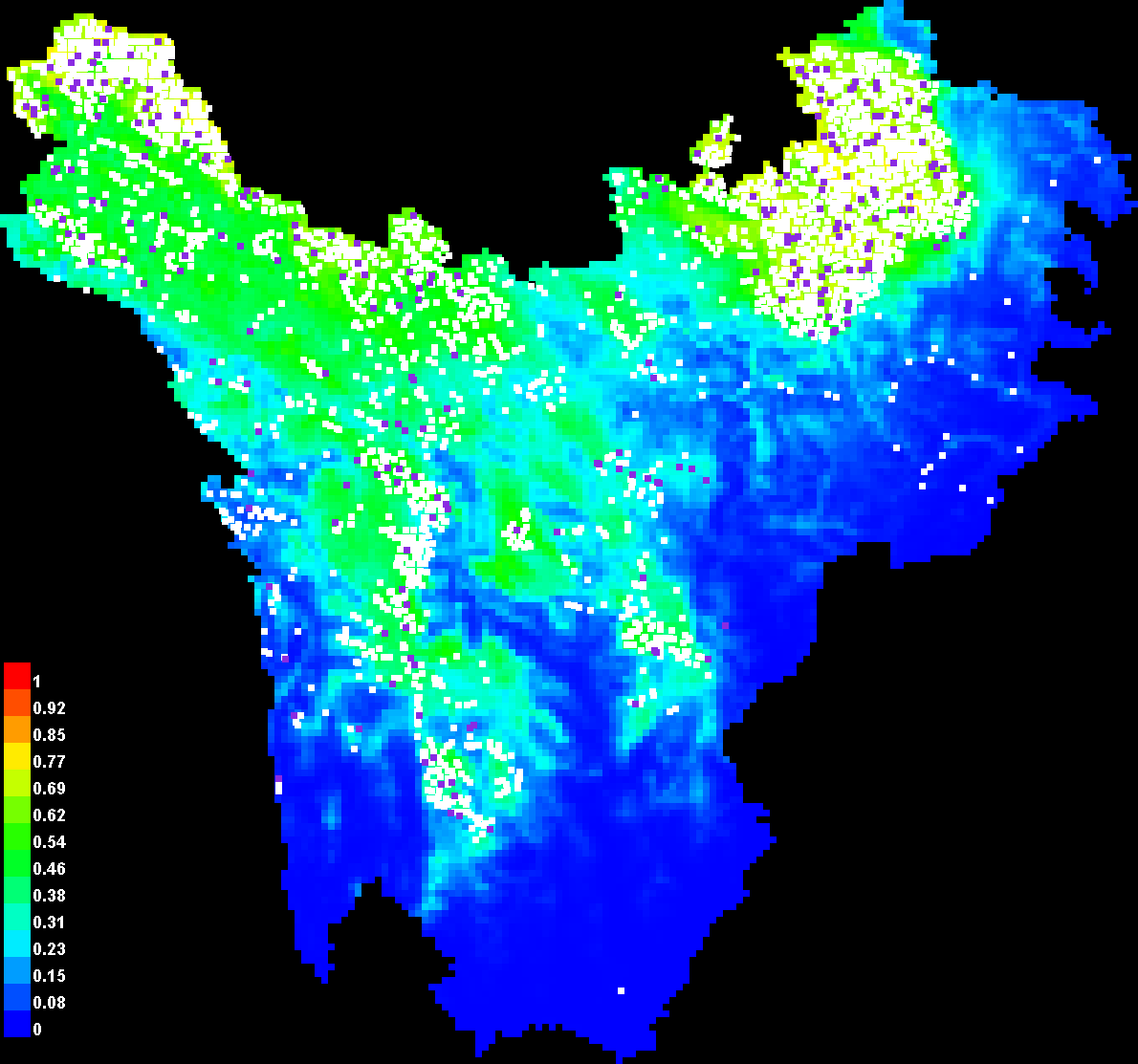

Supplement: Supplemental Information 1 [file peerj-12-18586-s001.zip › Maxent_data1/2.5mBIOssp126_2021-2040/RES/plots/wetland_7.png]

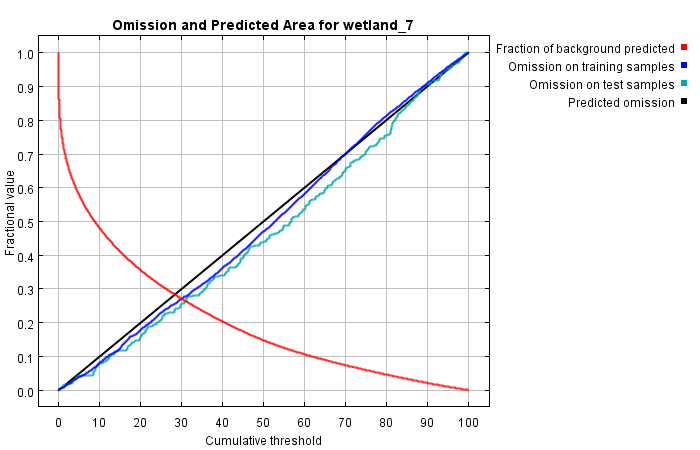

Supplement: Supplemental Information 1 [file peerj-12-18586-s001.zip › Maxent_data1/2.5mBIOssp126_2021-2040/RES/plots/wetland_7_omission.png]

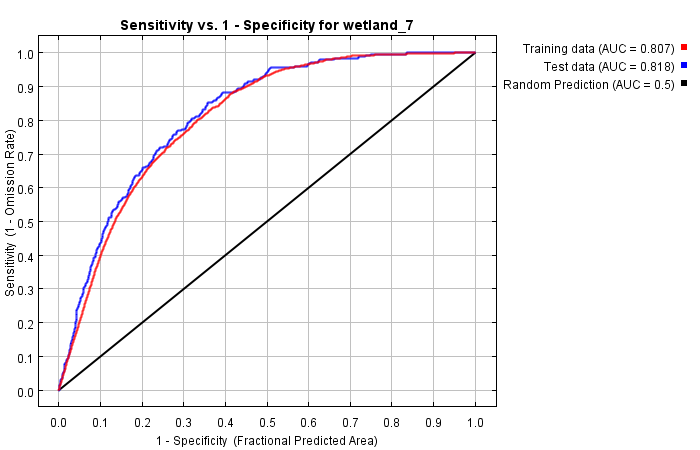

Supplement: Supplemental Information 1 [file peerj-12-18586-s001.zip › Maxent_data1/2.5mBIOssp126_2021-2040/RES/plots/wetland_7_roc.png]

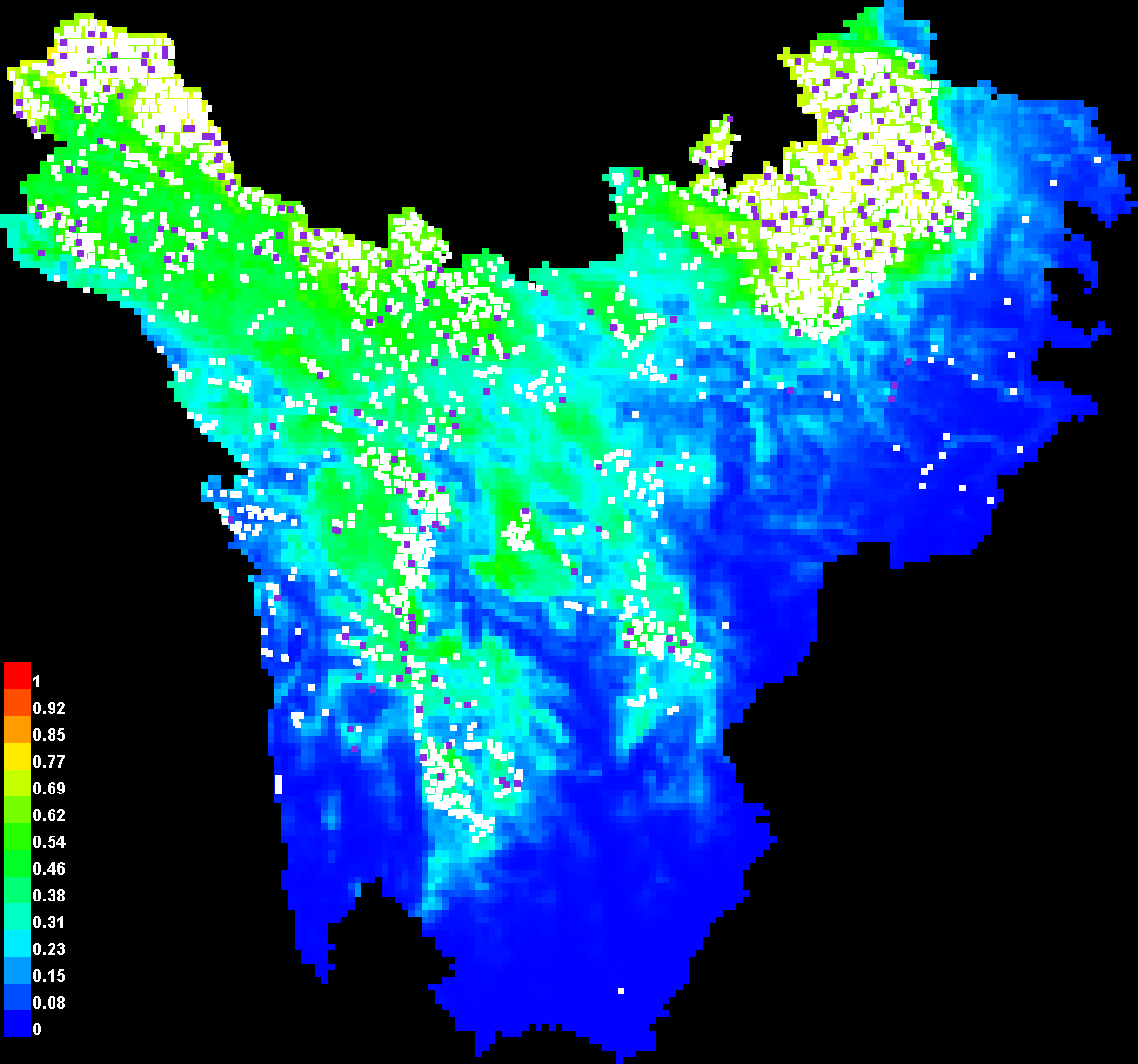

Supplement: Supplemental Information 1 [file peerj-12-18586-s001.zip › Maxent_data1/2.5mBIOssp126_2021-2040/RES/plots/wetland_8.png]

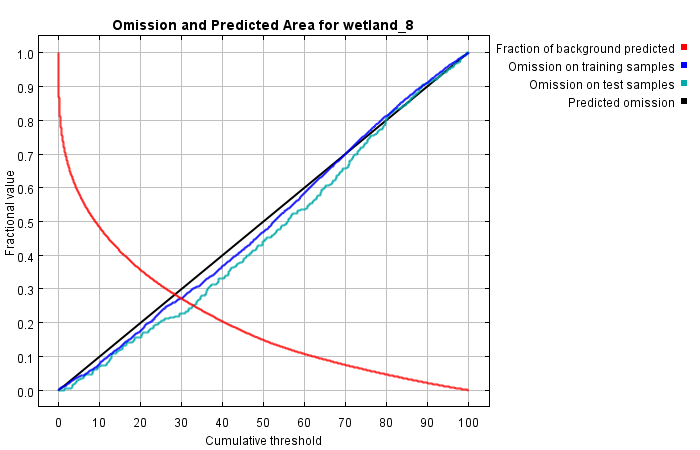

Supplement: Supplemental Information 1 [file peerj-12-18586-s001.zip › Maxent_data1/2.5mBIOssp126_2021-2040/RES/plots/wetland_8_omission.png]

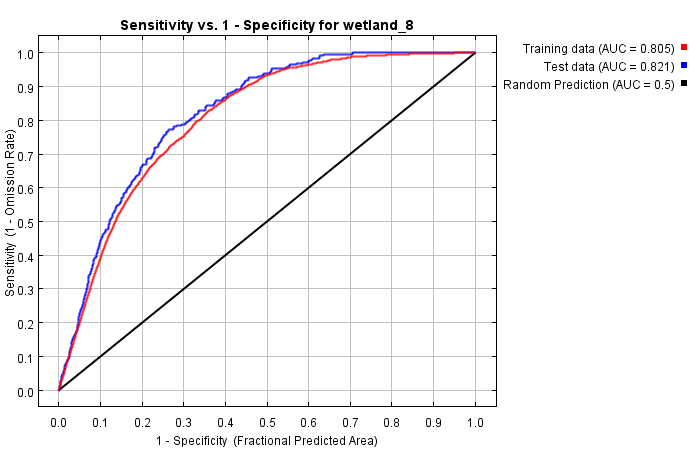

Supplement: Supplemental Information 1 [file peerj-12-18586-s001.zip › Maxent_data1/2.5mBIOssp126_2021-2040/RES/plots/wetland_8_roc.png]

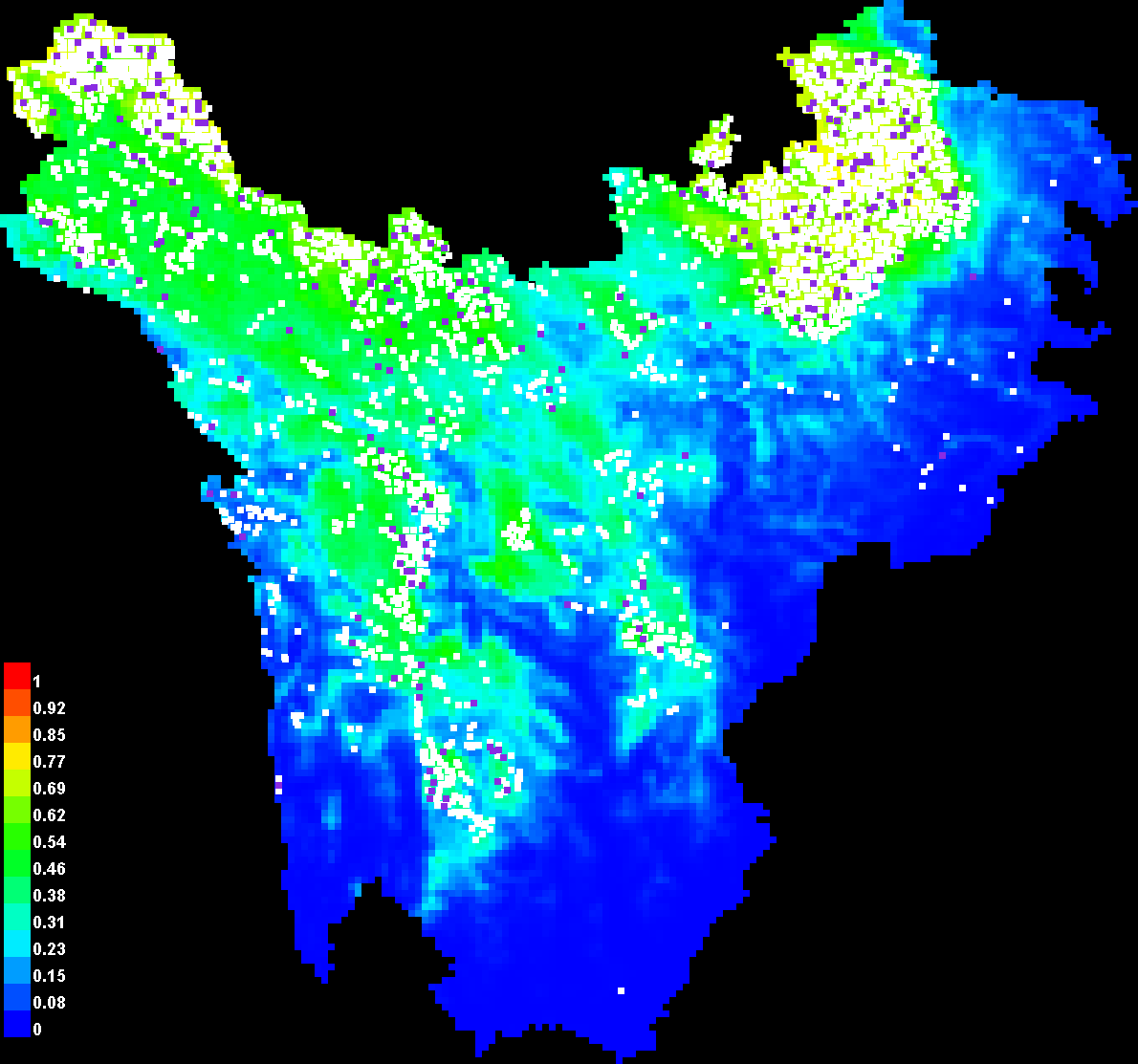

Supplement: Supplemental Information 1 [file peerj-12-18586-s001.zip › Maxent_data1/2.5mBIOssp126_2021-2040/RES/plots/wetland_9.png]

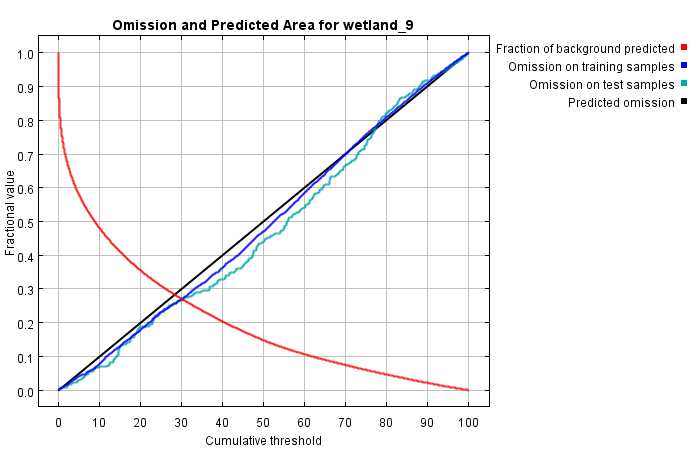

Supplement: Supplemental Information 1 [file peerj-12-18586-s001.zip › Maxent_data1/2.5mBIOssp126_2021-2040/RES/plots/wetland_9_omission.png]

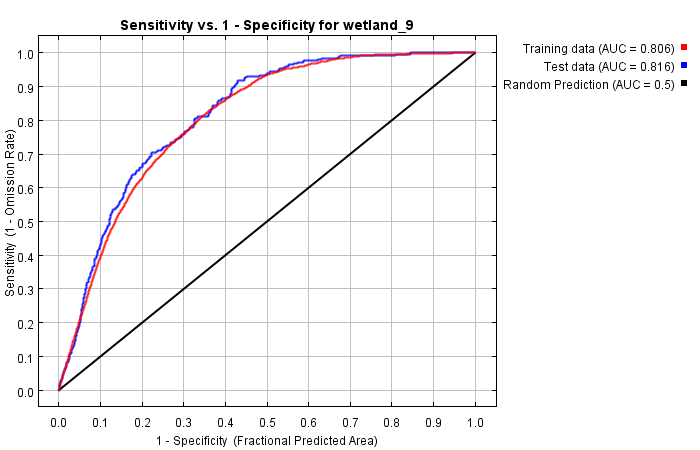

Supplement: Supplemental Information 1 [file peerj-12-18586-s001.zip › Maxent_data1/2.5mBIOssp126_2021-2040/RES/plots/wetland_9_roc.png]

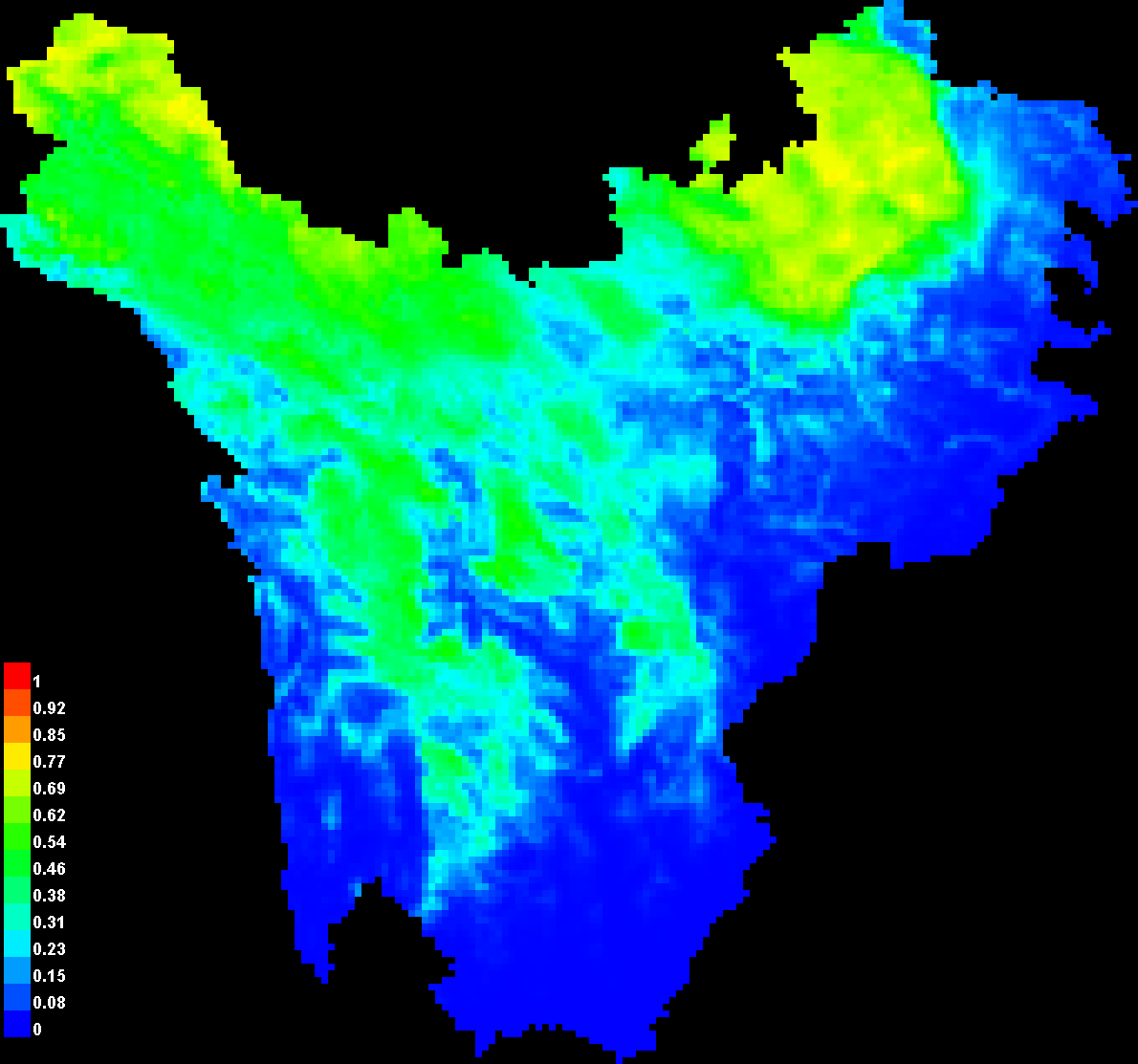

Supplement: Supplemental Information 1 [file peerj-12-18586-s001.zip › Maxent_data1/2.5mBIOssp126_2021-2040/RES/plots/wetland_avg.png]

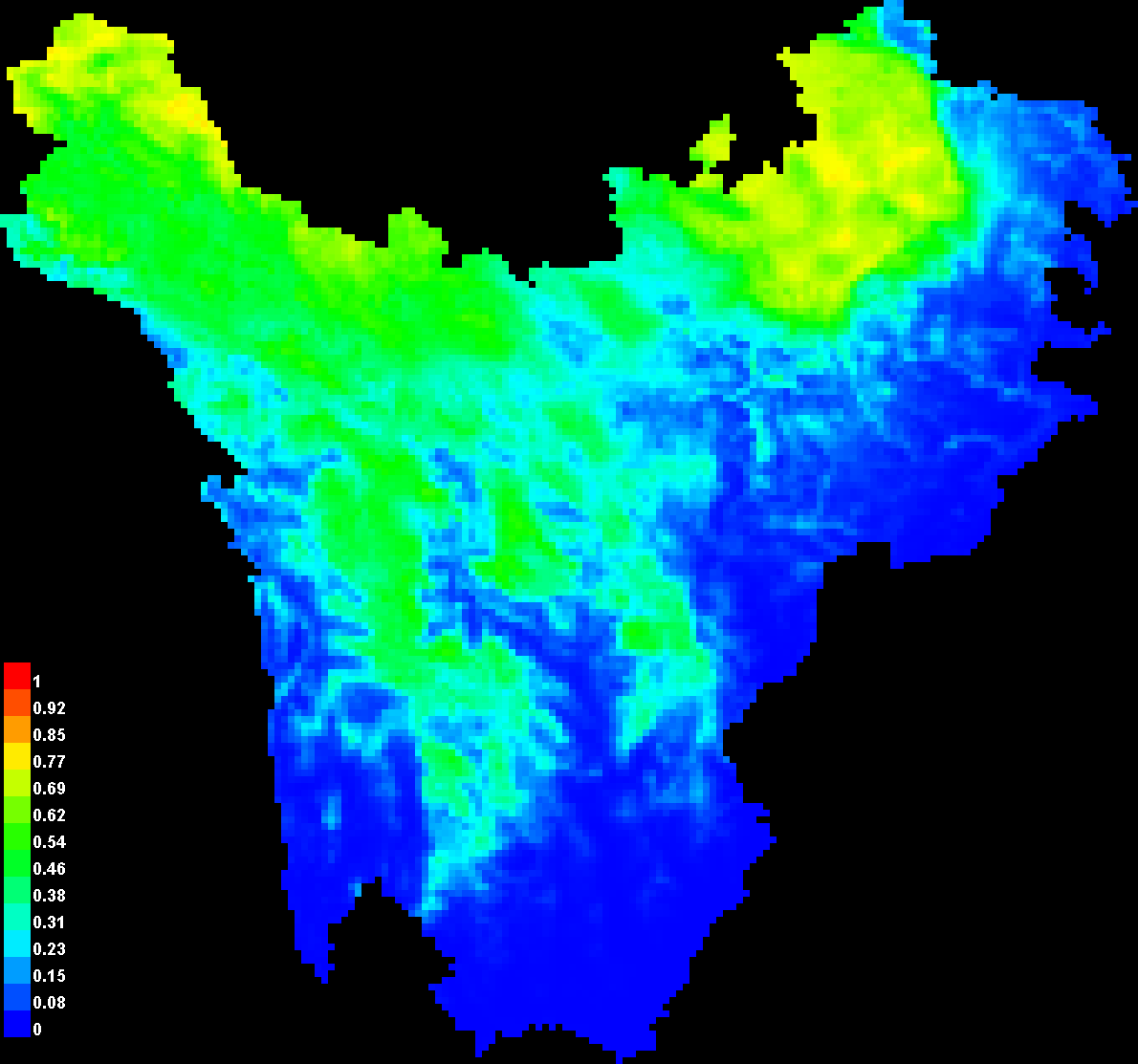

Supplement: Supplemental Information 1 [file peerj-12-18586-s001.zip › Maxent_data1/2.5mBIOssp126_2021-2040/RES/plots/wetland_max.png]

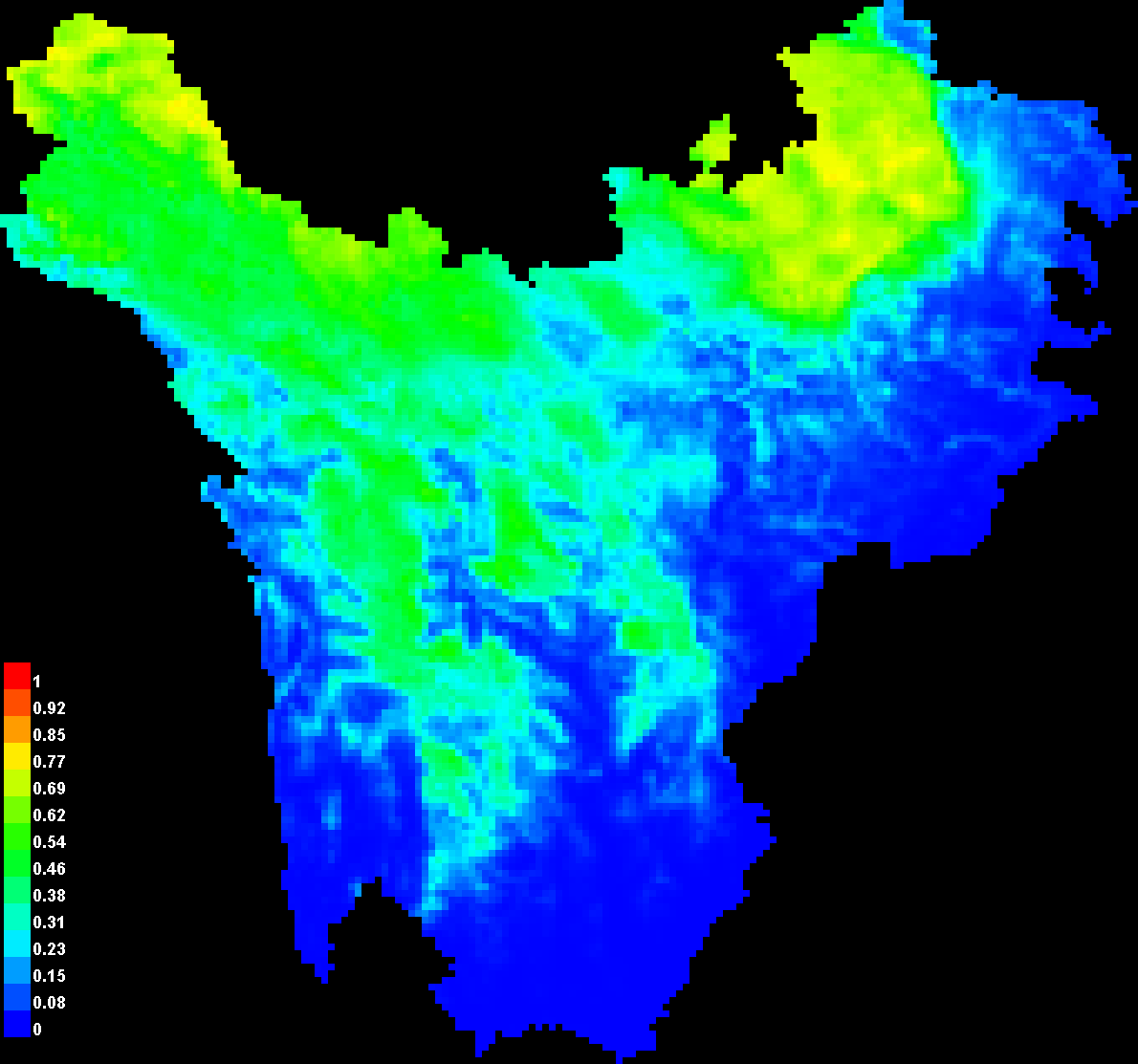

Supplement: Supplemental Information 1 [file peerj-12-18586-s001.zip › Maxent_data1/2.5mBIOssp126_2021-2040/RES/plots/wetland_median.png]

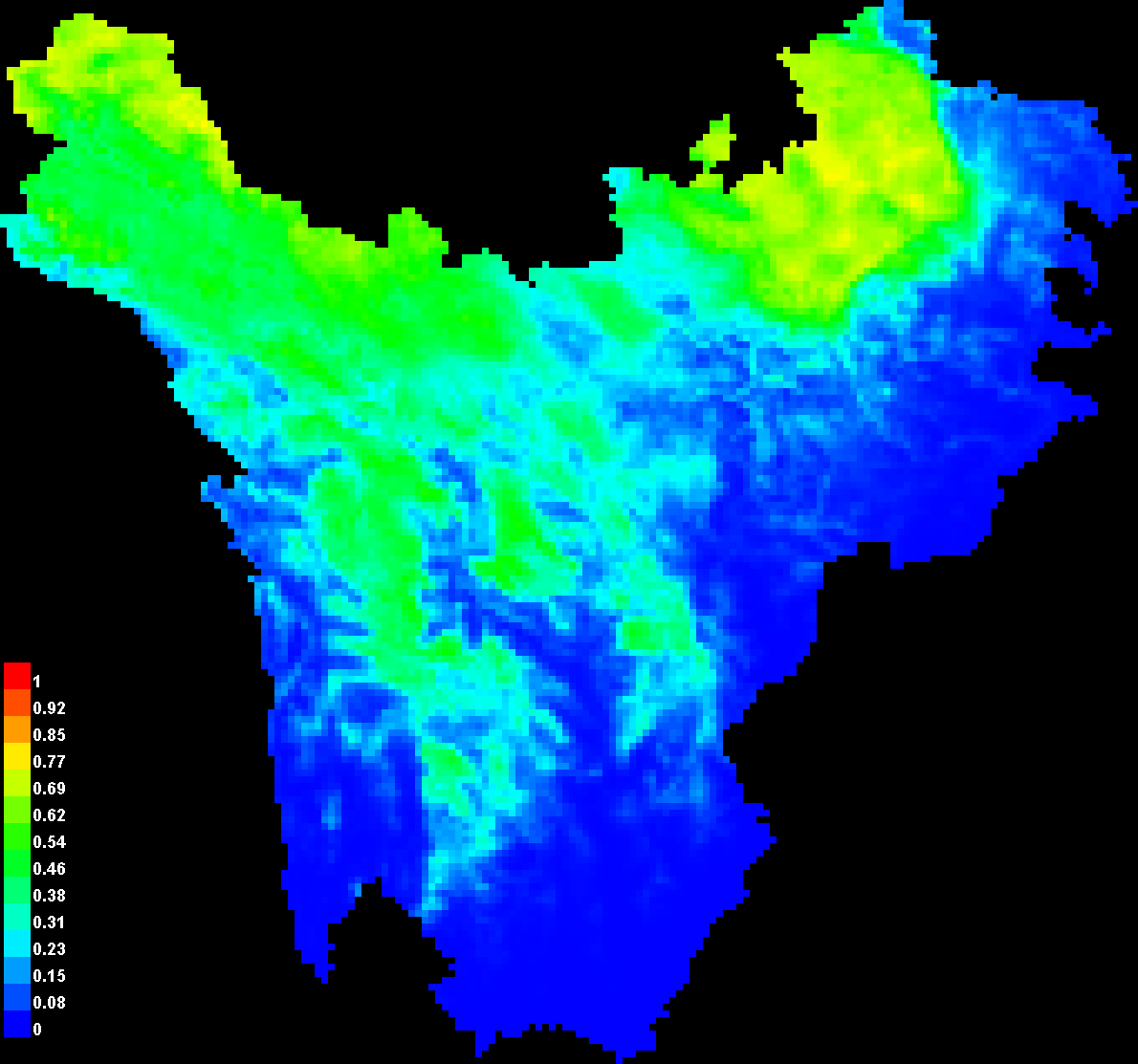

Supplement: Supplemental Information 1 [file peerj-12-18586-s001.zip › Maxent_data1/2.5mBIOssp126_2021-2040/RES/plots/wetland_min.png]

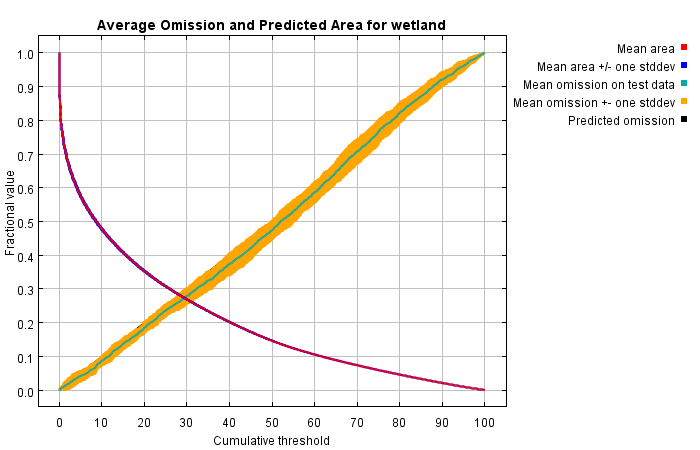

Supplement: Supplemental Information 1 [file peerj-12-18586-s001.zip › Maxent_data1/2.5mBIOssp126_2021-2040/RES/plots/wetland_omission.png]

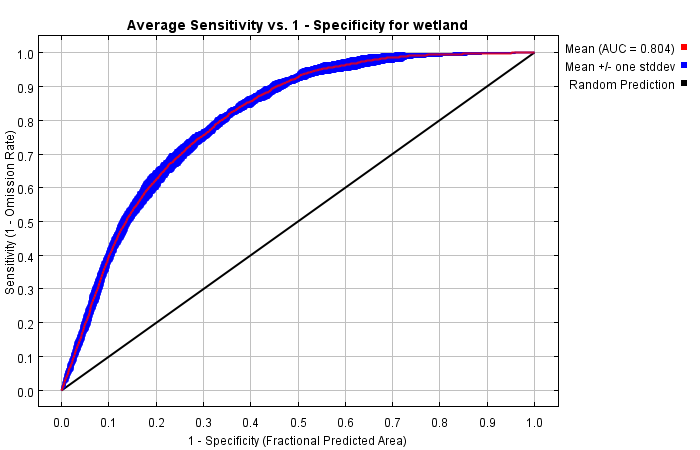

Supplement: Supplemental Information 1 [file peerj-12-18586-s001.zip › Maxent_data1/2.5mBIOssp126_2021-2040/RES/plots/wetland_roc.png]

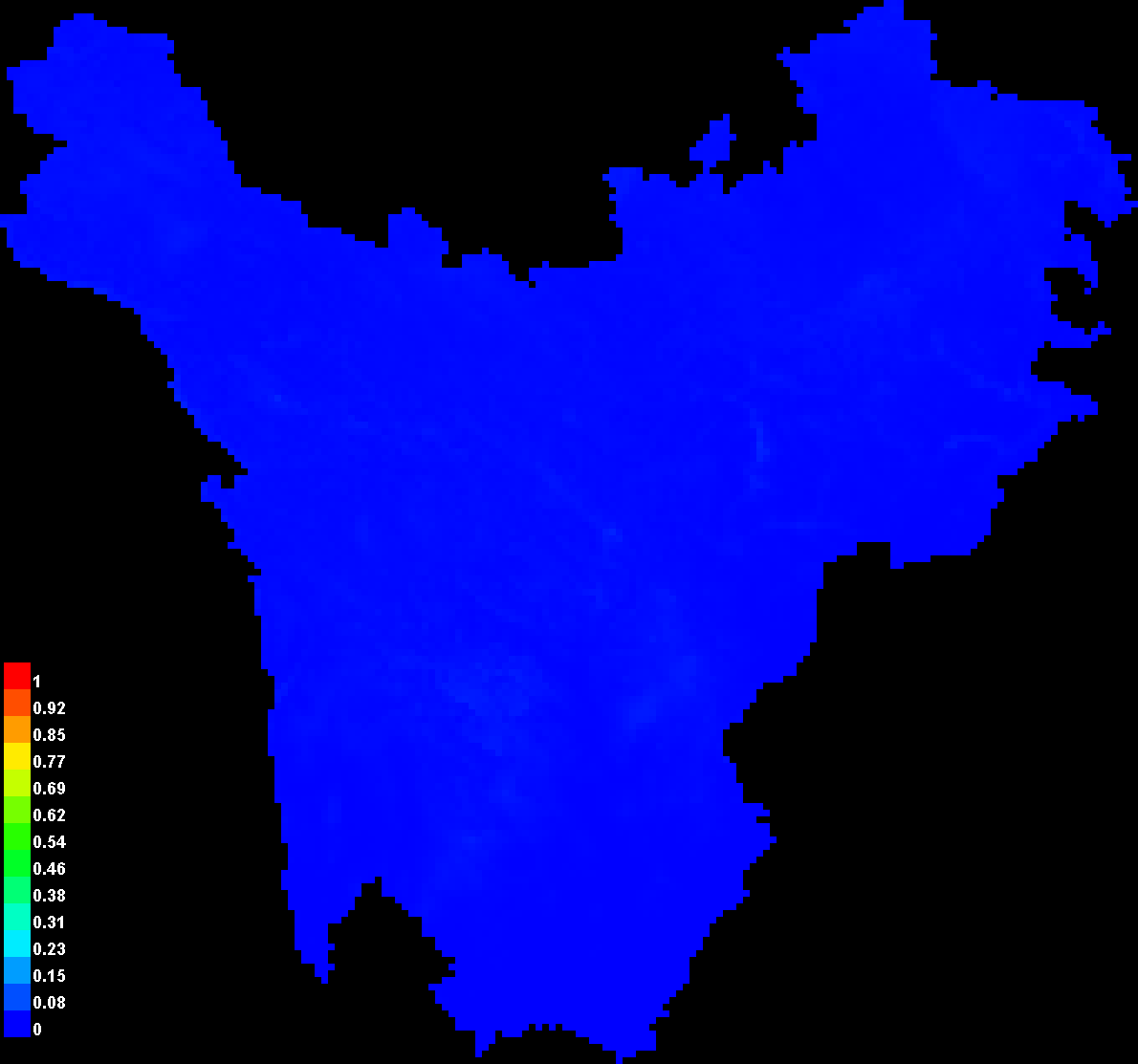

Supplement: Supplemental Information 1 [file peerj-12-18586-s001.zip › Maxent_data1/2.5mBIOssp126_2021-2040/RES/plots/wetland_stddev.png]

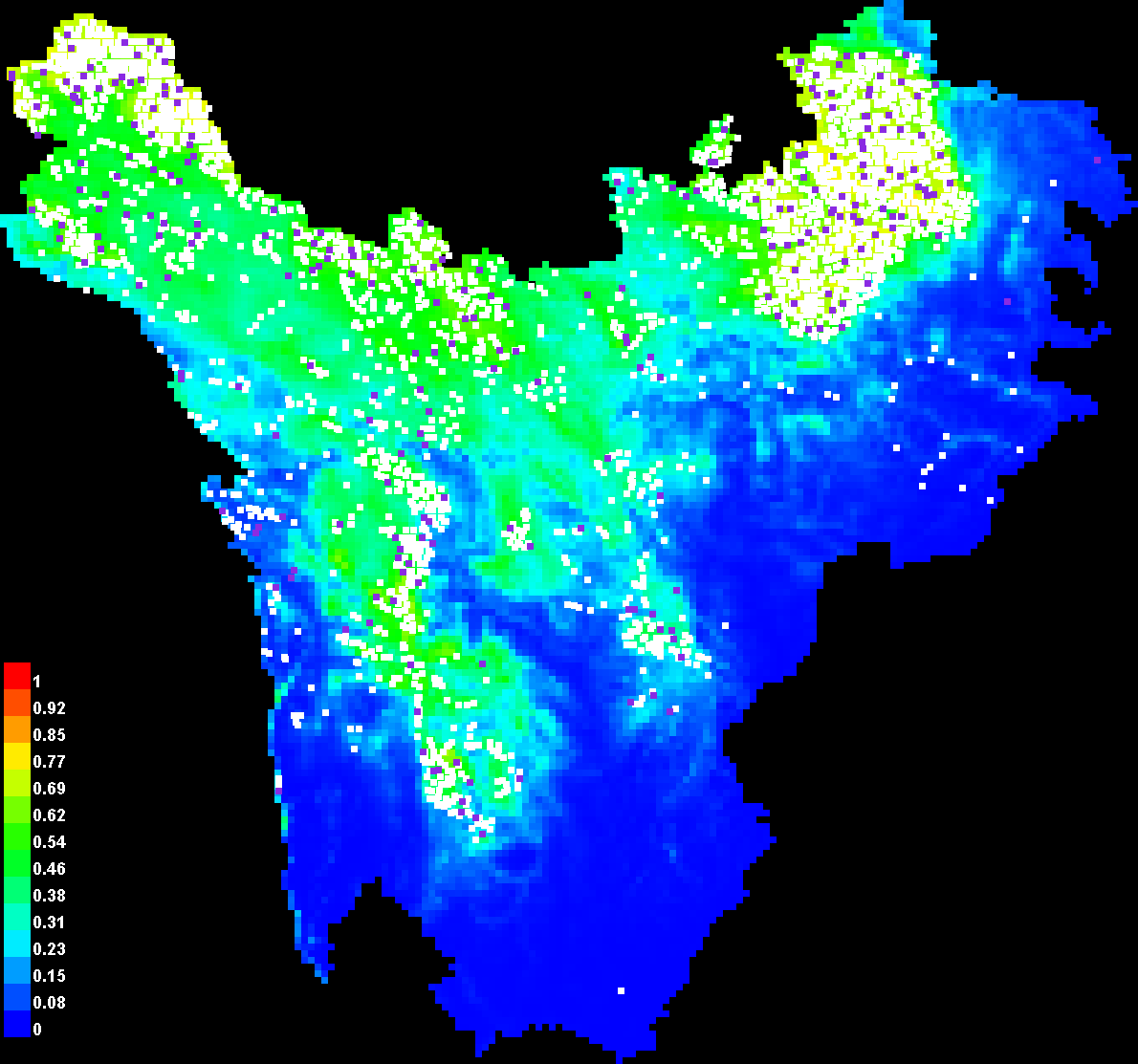

Supplement: Supplemental Information 2 [file peerj-12-18586-s002.zip › Maxent_data2/2.5mBIOssp245_2081-2100/RES/plots/wetland_0.png]

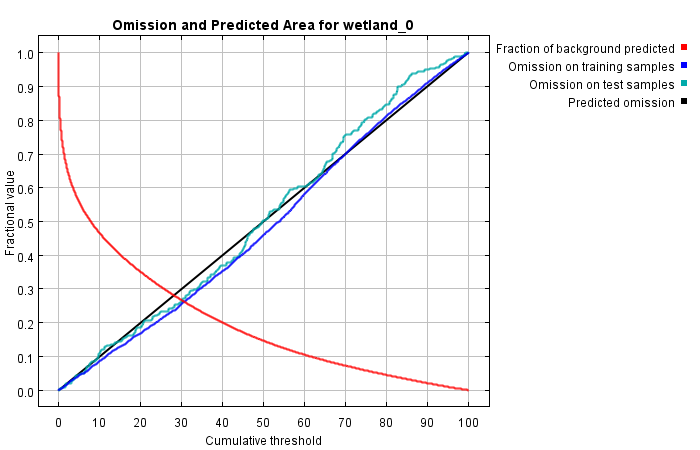

Supplement: Supplemental Information 2 [file peerj-12-18586-s002.zip › Maxent_data2/2.5mBIOssp245_2081-2100/RES/plots/wetland_0_omission.png]

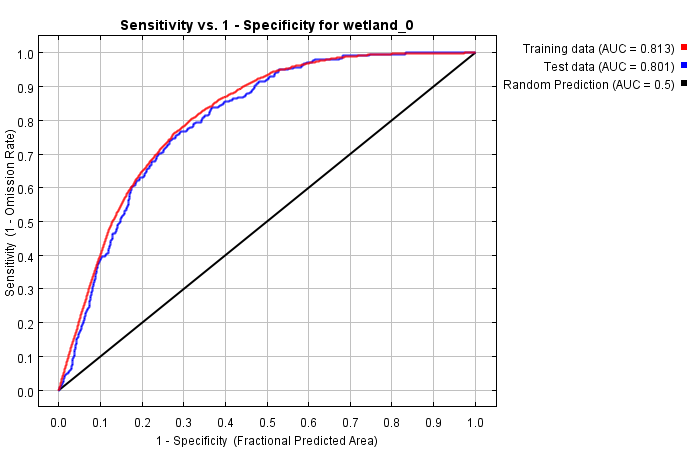

Supplement: Supplemental Information 2 [file peerj-12-18586-s002.zip › Maxent_data2/2.5mBIOssp245_2081-2100/RES/plots/wetland_0_roc.png]

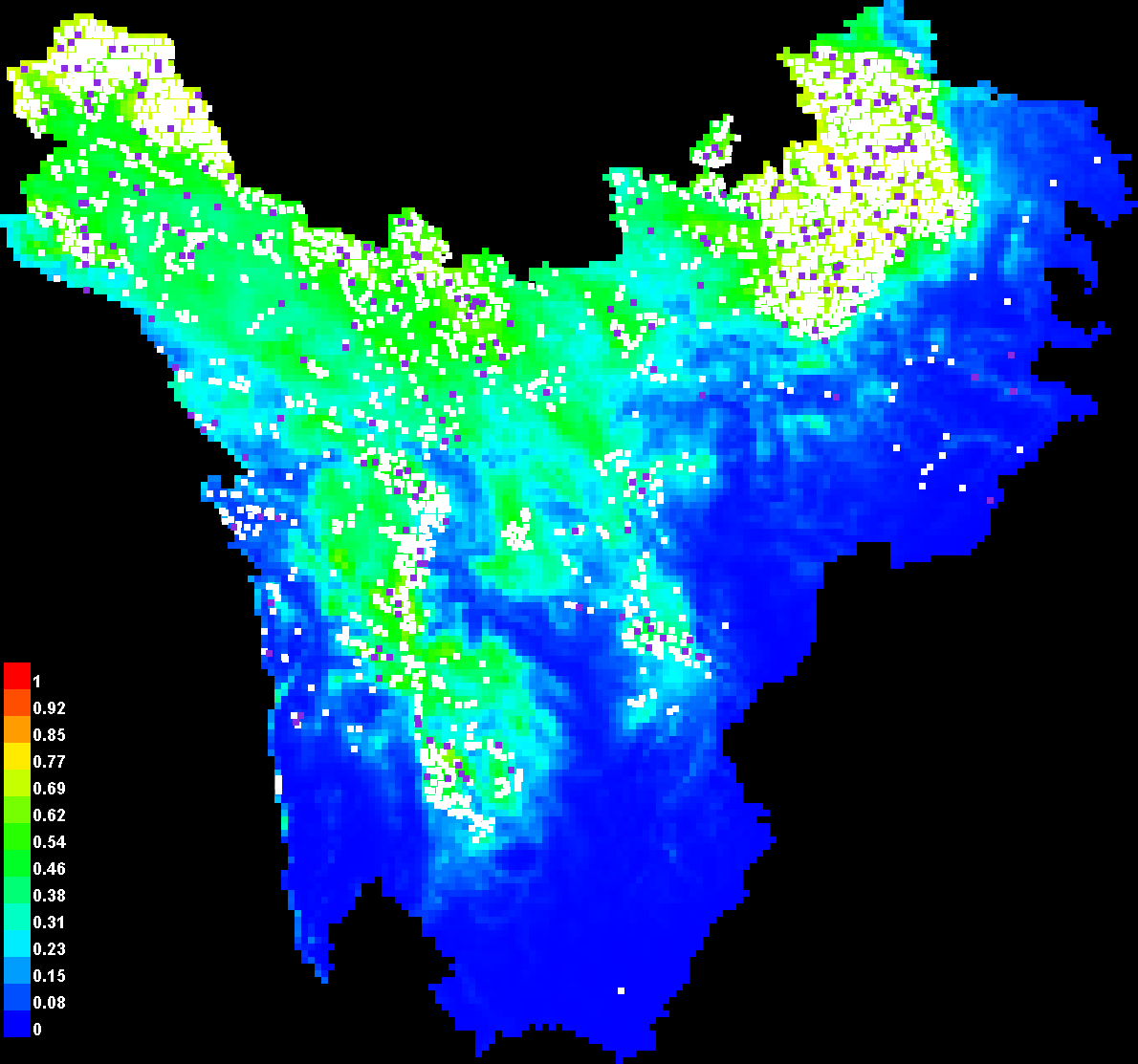

Supplement: Supplemental Information 2 [file peerj-12-18586-s002.zip › Maxent_data2/2.5mBIOssp245_2081-2100/RES/plots/wetland_1.png]

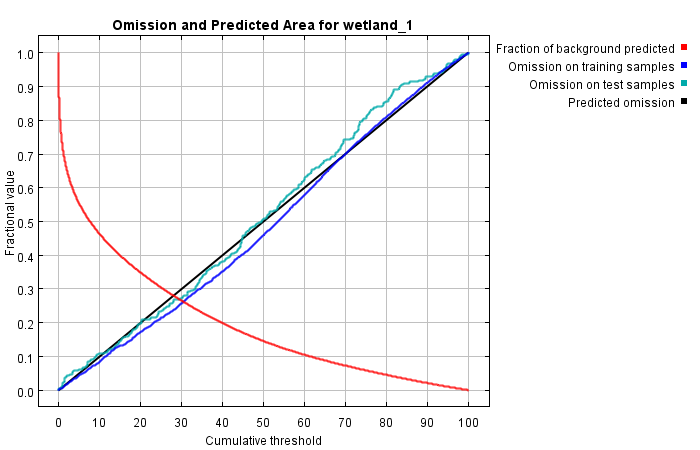

Supplement: Supplemental Information 2 [file peerj-12-18586-s002.zip › Maxent_data2/2.5mBIOssp245_2081-2100/RES/plots/wetland_1_omission.png]

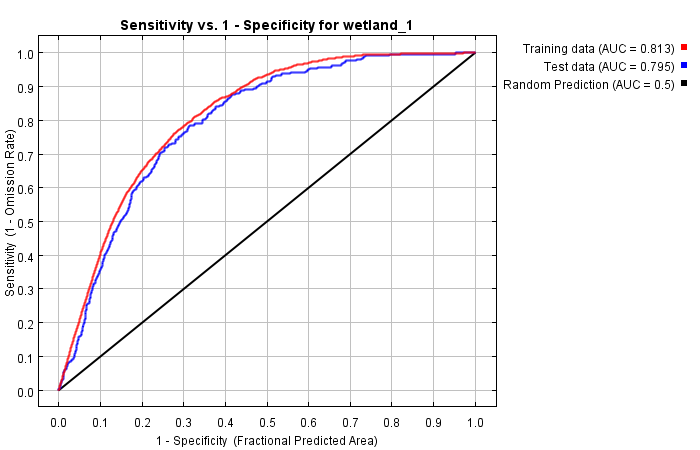

Supplement: Supplemental Information 2 [file peerj-12-18586-s002.zip › Maxent_data2/2.5mBIOssp245_2081-2100/RES/plots/wetland_1_roc.png]

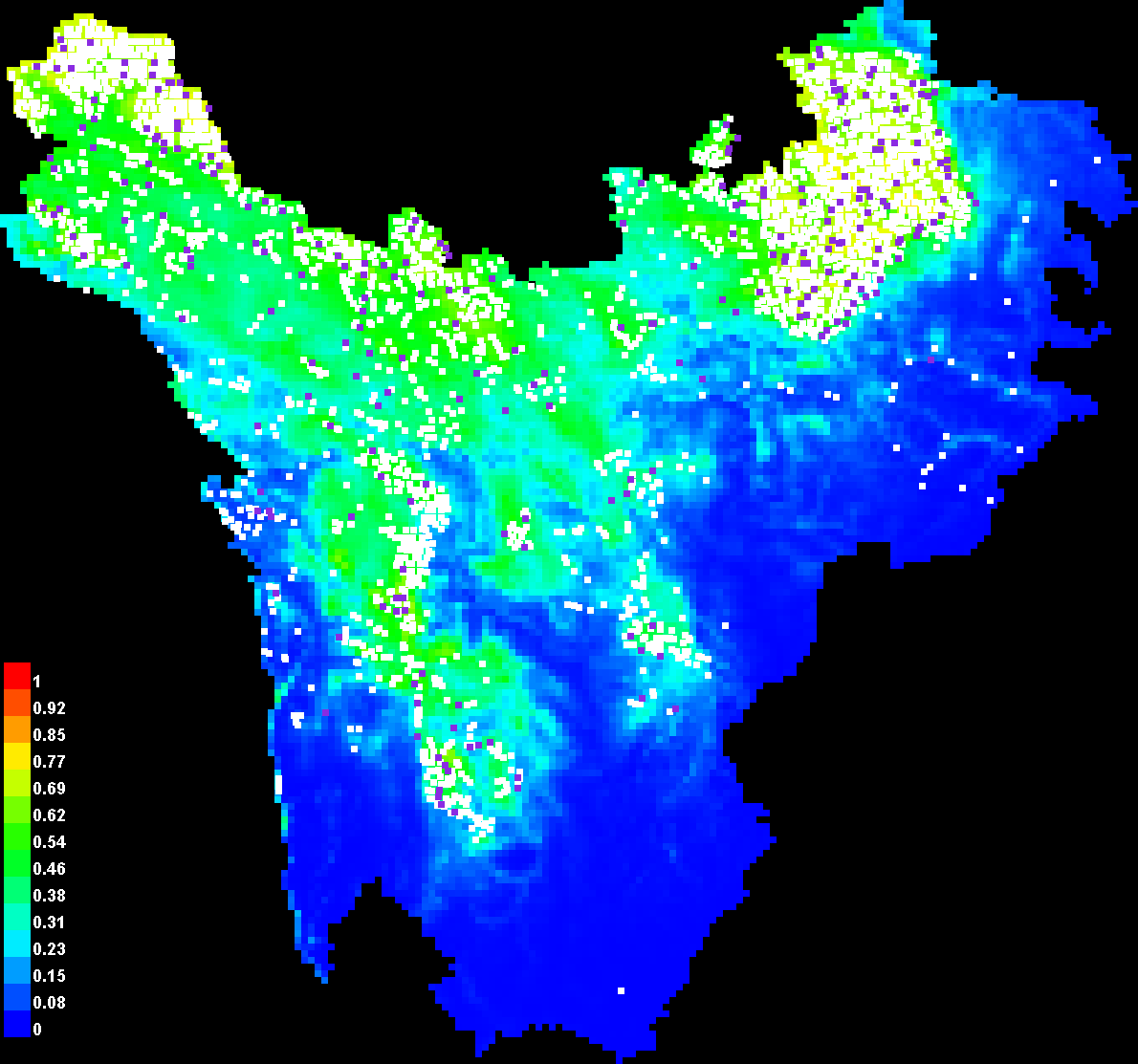

Supplement: Supplemental Information 2 [file peerj-12-18586-s002.zip › Maxent_data2/2.5mBIOssp245_2081-2100/RES/plots/wetland_2.png]

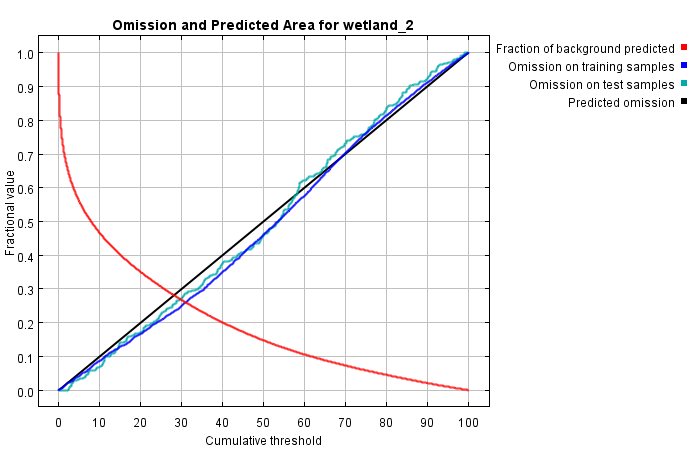

Supplement: Supplemental Information 2 [file peerj-12-18586-s002.zip › Maxent_data2/2.5mBIOssp245_2081-2100/RES/plots/wetland_2_omission.png]

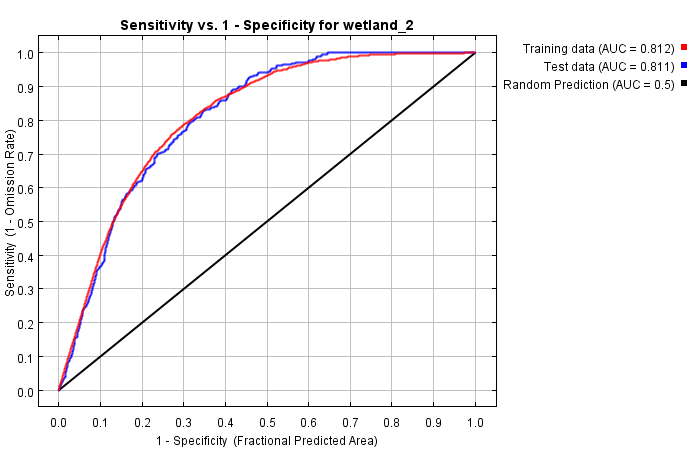

Supplement: Supplemental Information 2 [file peerj-12-18586-s002.zip › Maxent_data2/2.5mBIOssp245_2081-2100/RES/plots/wetland_2_roc.png]

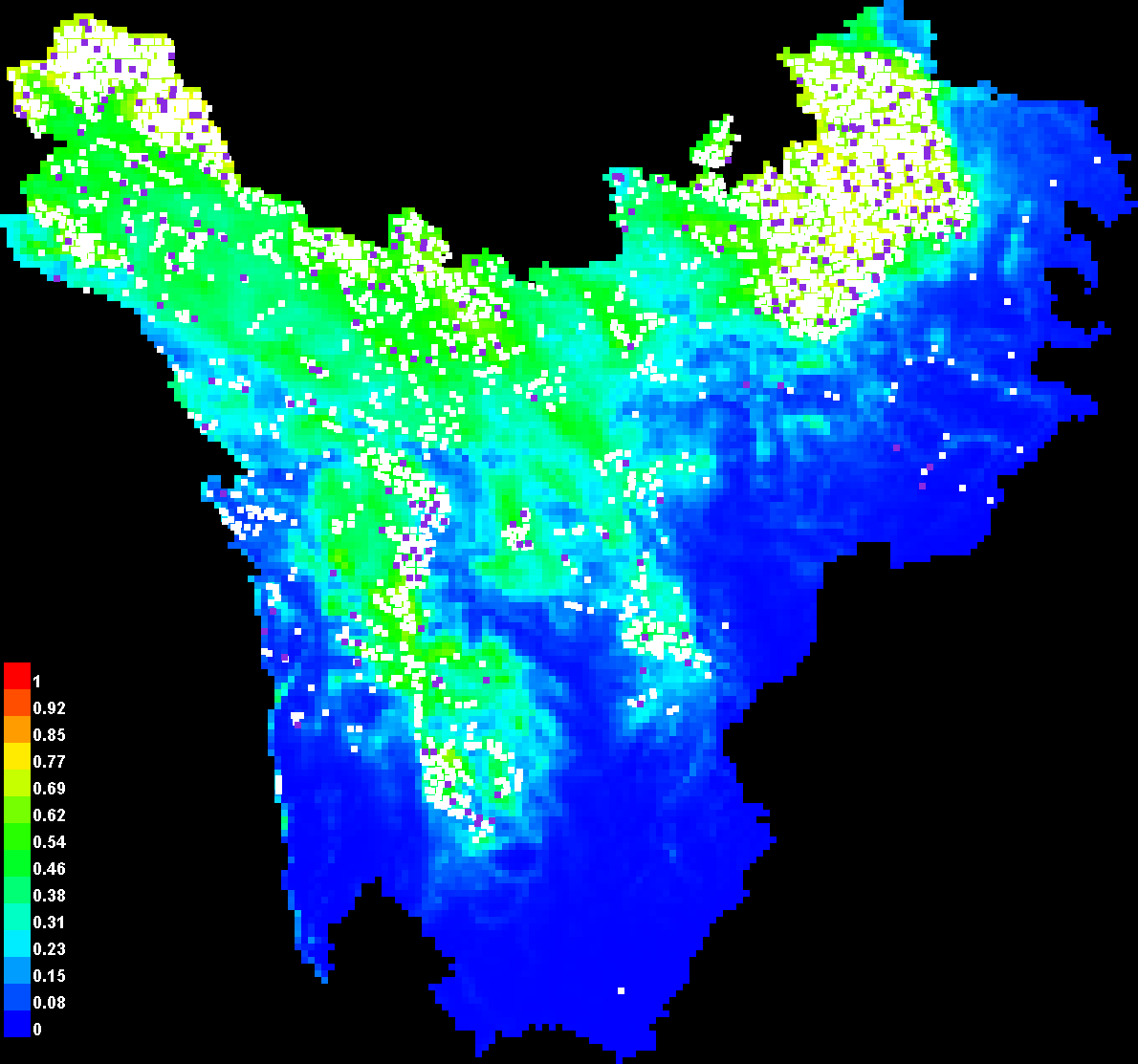

Supplement: Supplemental Information 2 [file peerj-12-18586-s002.zip › Maxent_data2/2.5mBIOssp245_2081-2100/RES/plots/wetland_3.png]

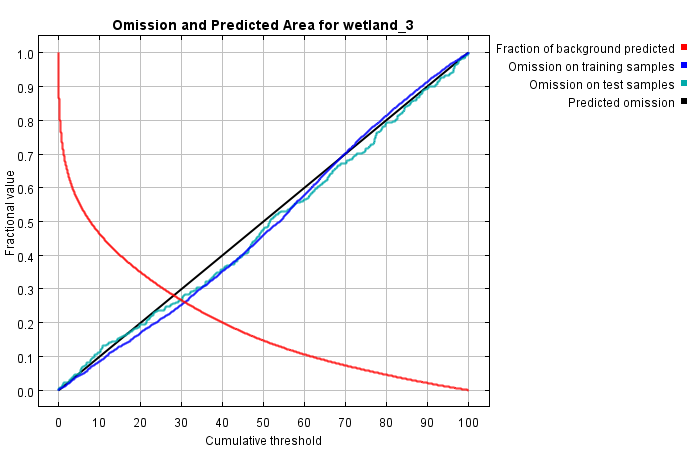

Supplement: Supplemental Information 2 [file peerj-12-18586-s002.zip › Maxent_data2/2.5mBIOssp245_2081-2100/RES/plots/wetland_3_omission.png]

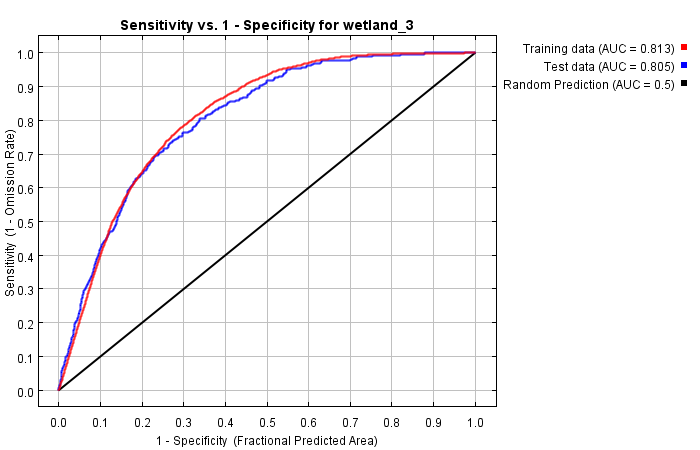

Supplement: Supplemental Information 2 [file peerj-12-18586-s002.zip › Maxent_data2/2.5mBIOssp245_2081-2100/RES/plots/wetland_3_roc.png]

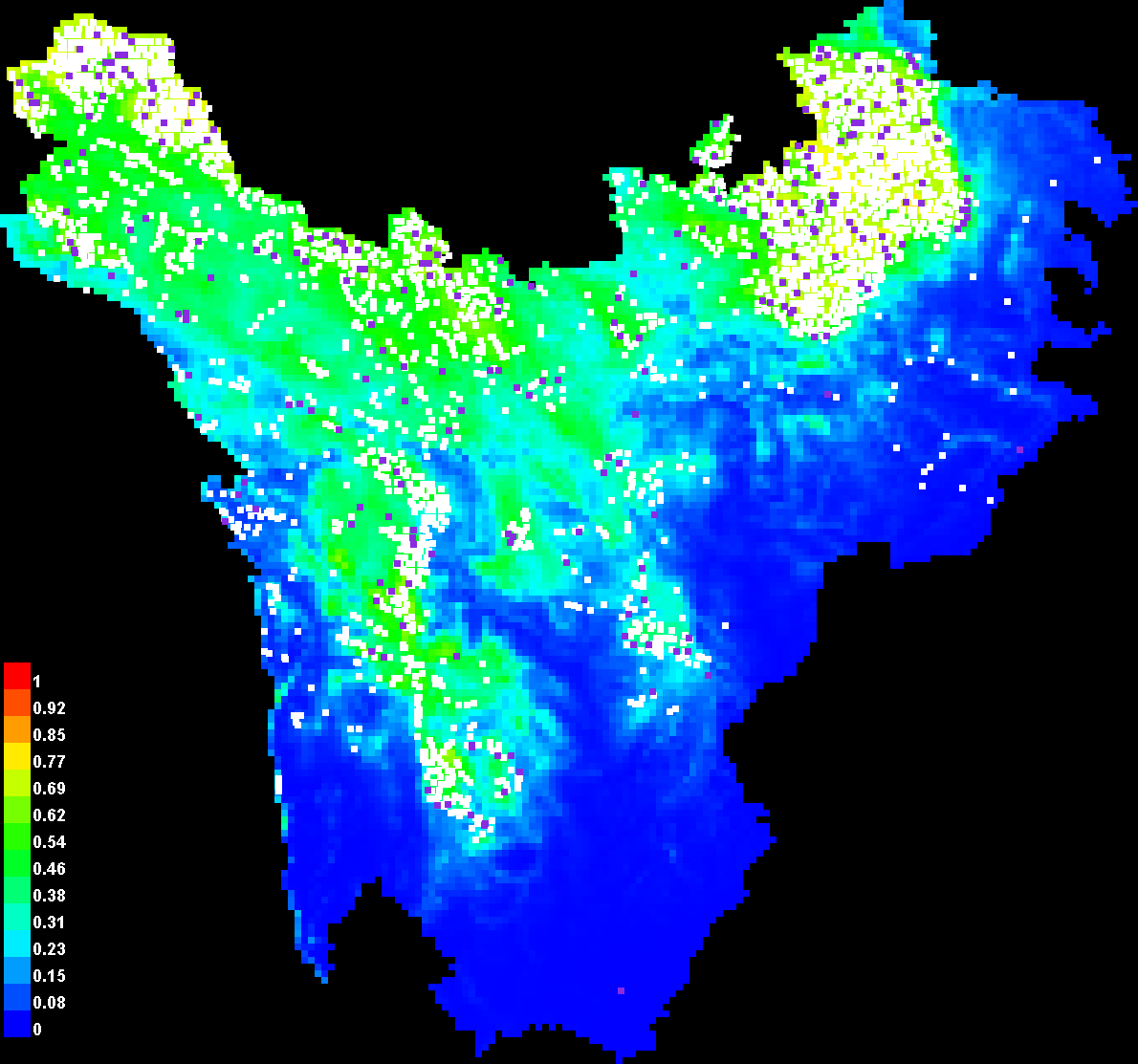

Supplement: Supplemental Information 2 [file peerj-12-18586-s002.zip › Maxent_data2/2.5mBIOssp245_2081-2100/RES/plots/wetland_4.png]

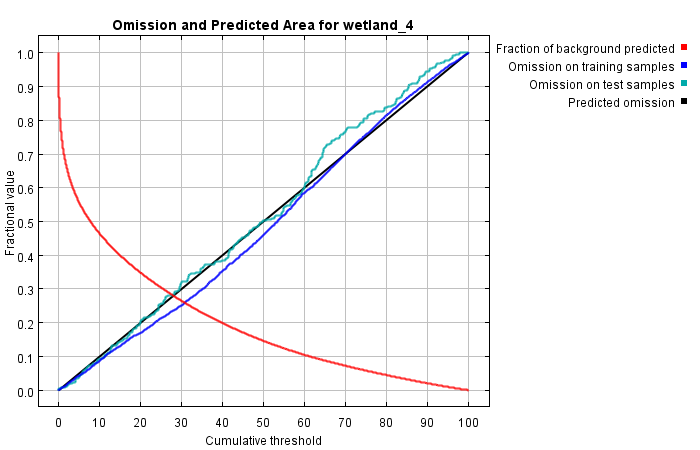

Supplement: Supplemental Information 2 [file peerj-12-18586-s002.zip › Maxent_data2/2.5mBIOssp245_2081-2100/RES/plots/wetland_4_omission.png]

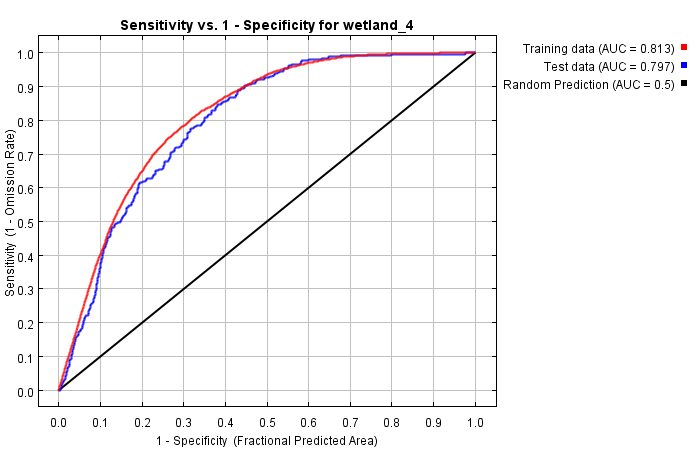

Supplement: Supplemental Information 2 [file peerj-12-18586-s002.zip › Maxent_data2/2.5mBIOssp245_2081-2100/RES/plots/wetland_4_roc.png]

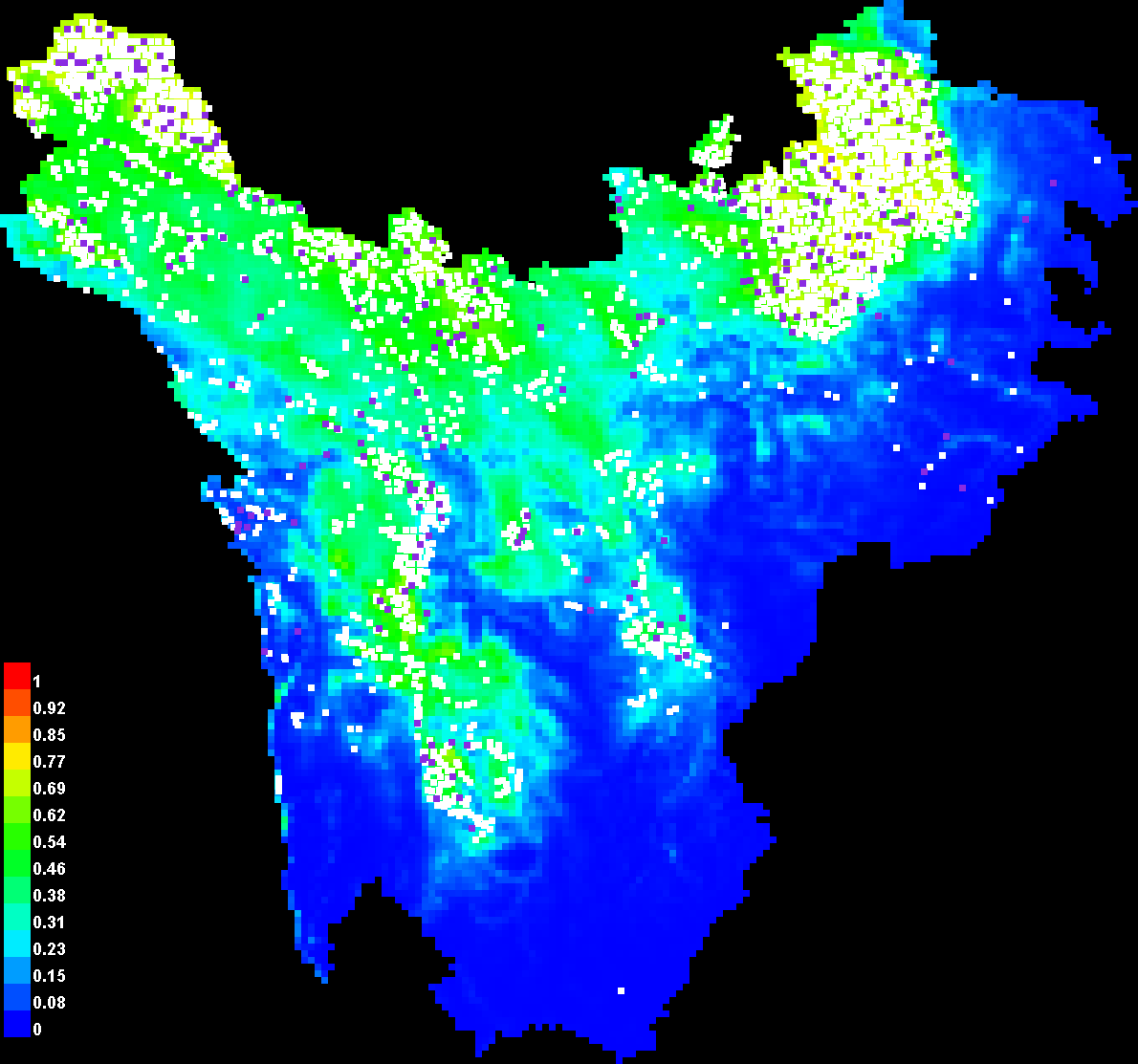

Supplement: Supplemental Information 2 [file peerj-12-18586-s002.zip › Maxent_data2/2.5mBIOssp245_2081-2100/RES/plots/wetland_5.png]

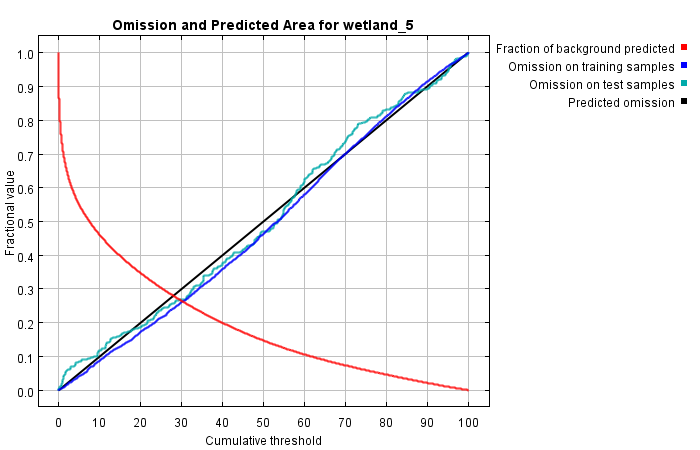

Supplement: Supplemental Information 2 [file peerj-12-18586-s002.zip › Maxent_data2/2.5mBIOssp245_2081-2100/RES/plots/wetland_5_omission.png]

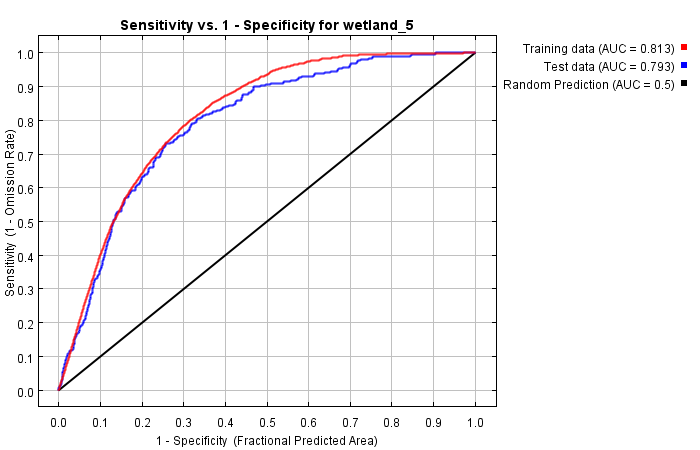

Supplement: Supplemental Information 2 [file peerj-12-18586-s002.zip › Maxent_data2/2.5mBIOssp245_2081-2100/RES/plots/wetland_5_roc.png]

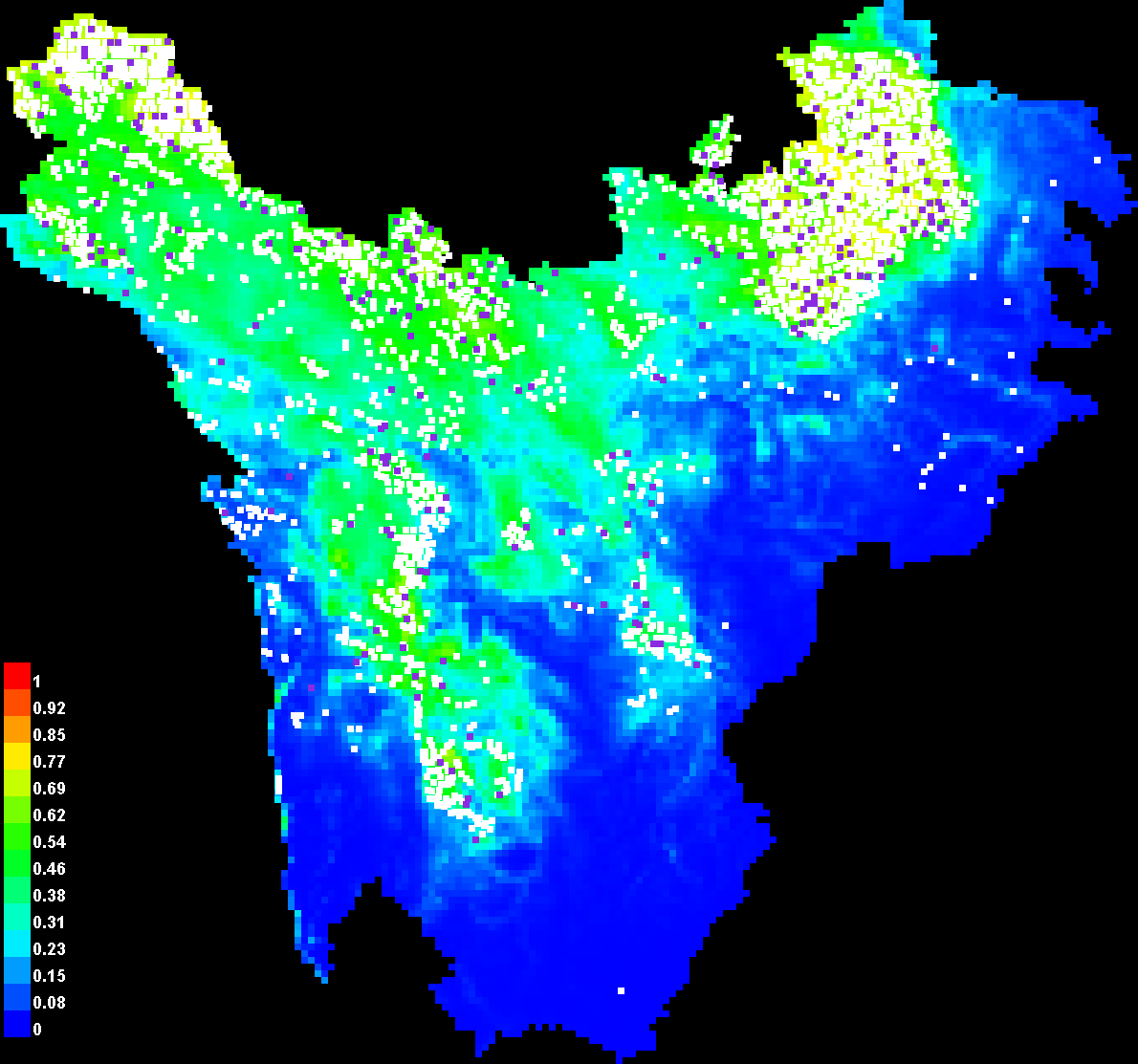

Supplement: Supplemental Information 2 [file peerj-12-18586-s002.zip › Maxent_data2/2.5mBIOssp245_2081-2100/RES/plots/wetland_6.png]

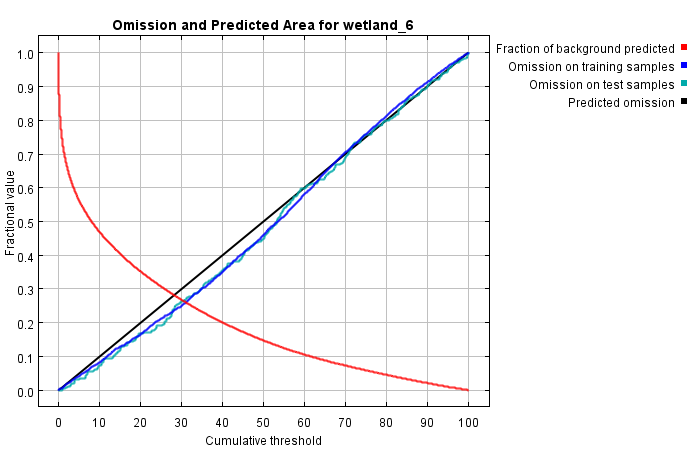

Supplement: Supplemental Information 2 [file peerj-12-18586-s002.zip › Maxent_data2/2.5mBIOssp245_2081-2100/RES/plots/wetland_6_omission.png]

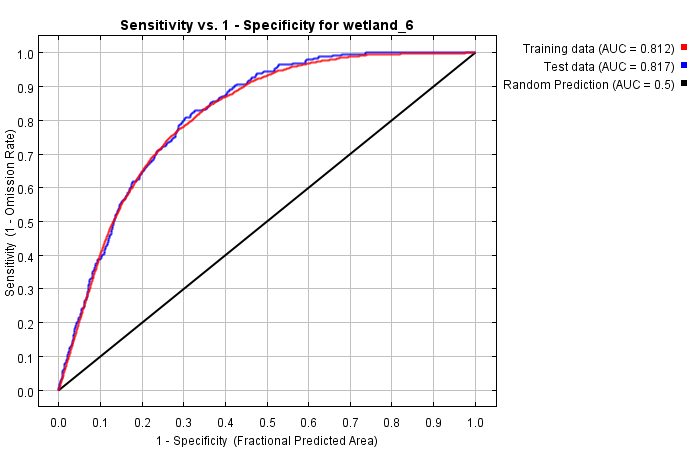

Supplement: Supplemental Information 2 [file peerj-12-18586-s002.zip › Maxent_data2/2.5mBIOssp245_2081-2100/RES/plots/wetland_6_roc.png]

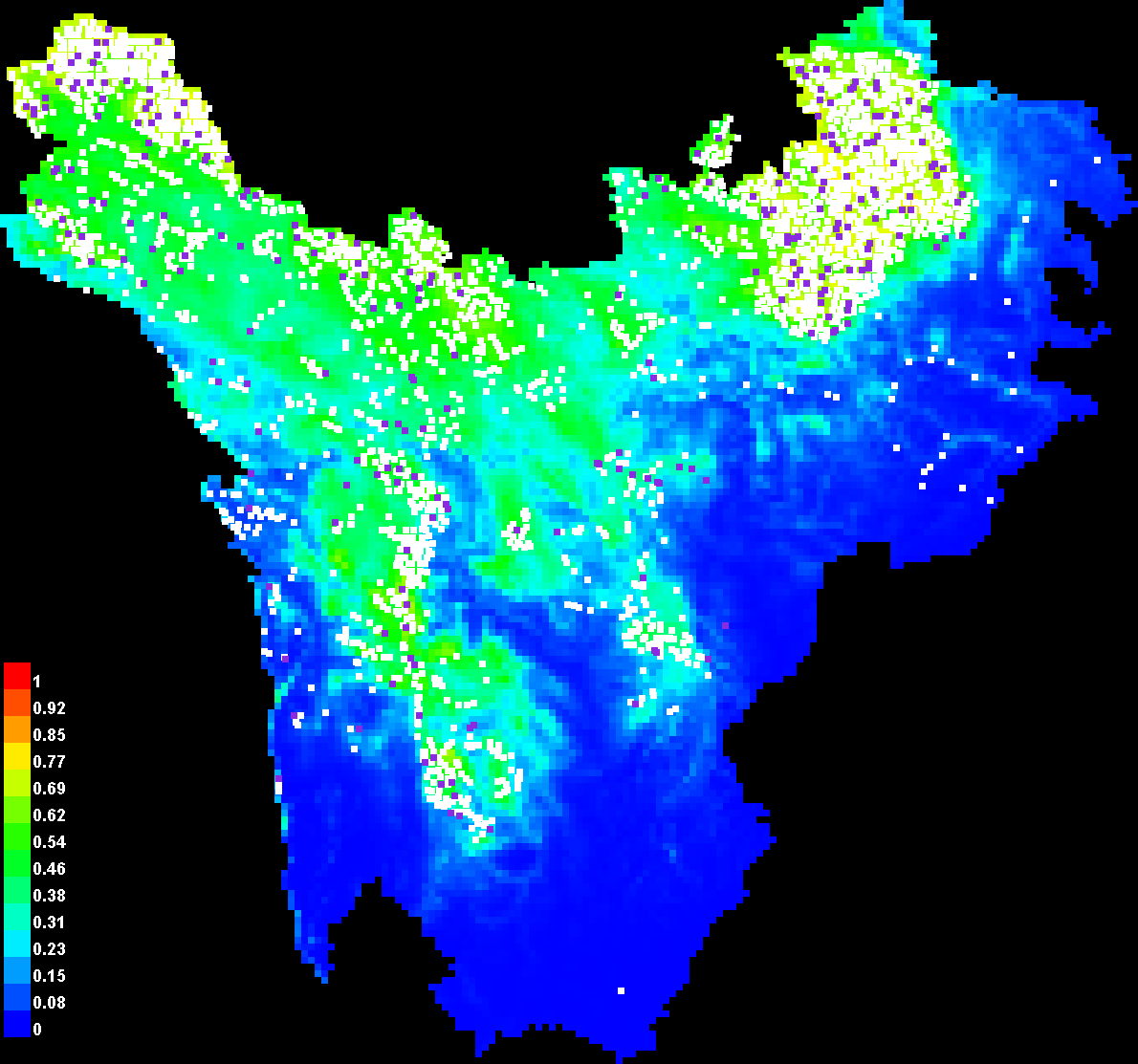

Supplement: Supplemental Information 2 [file peerj-12-18586-s002.zip › Maxent_data2/2.5mBIOssp245_2081-2100/RES/plots/wetland_7.png]

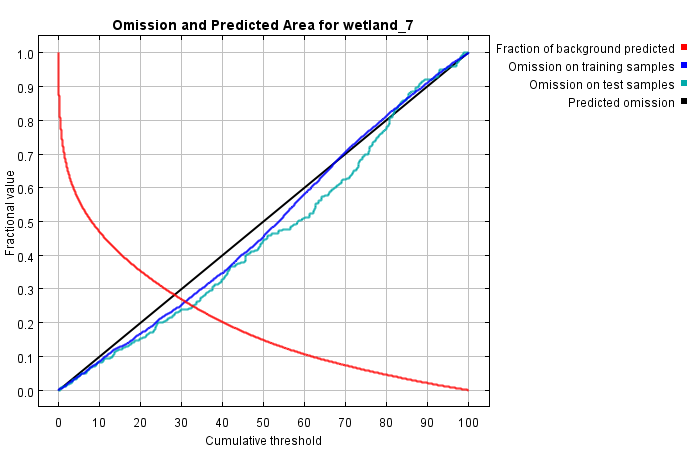

Supplement: Supplemental Information 2 [file peerj-12-18586-s002.zip › Maxent_data2/2.5mBIOssp245_2081-2100/RES/plots/wetland_7_omission.png]

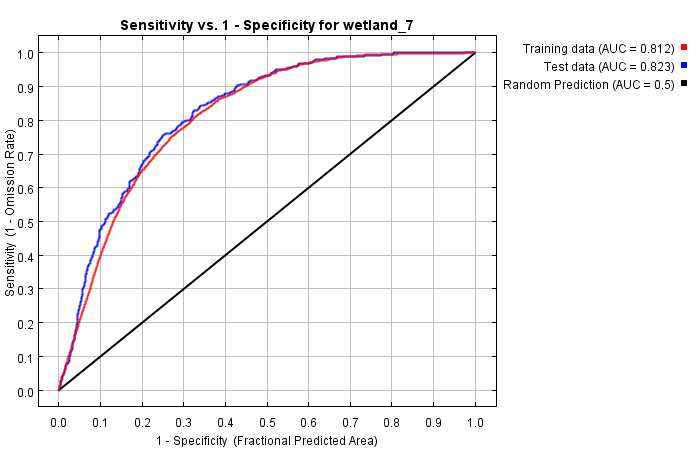

Supplement: Supplemental Information 2 [file peerj-12-18586-s002.zip › Maxent_data2/2.5mBIOssp245_2081-2100/RES/plots/wetland_7_roc.png]

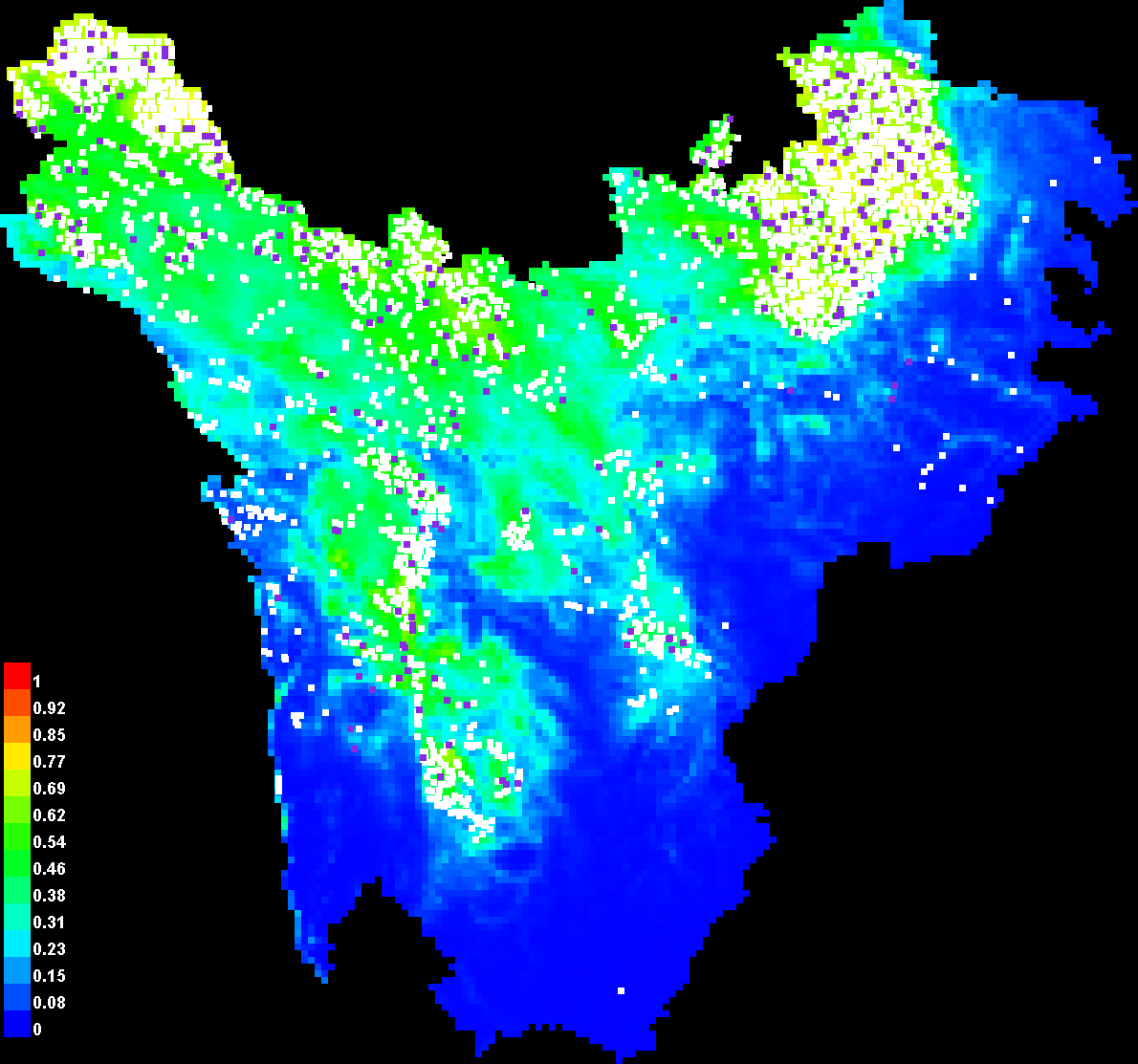

Supplement: Supplemental Information 2 [file peerj-12-18586-s002.zip › Maxent_data2/2.5mBIOssp245_2081-2100/RES/plots/wetland_8.png]

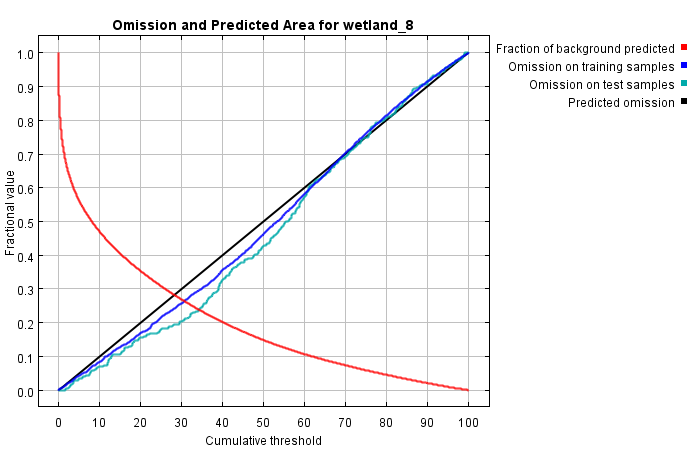

Supplement: Supplemental Information 2 [file peerj-12-18586-s002.zip › Maxent_data2/2.5mBIOssp245_2081-2100/RES/plots/wetland_8_omission.png]

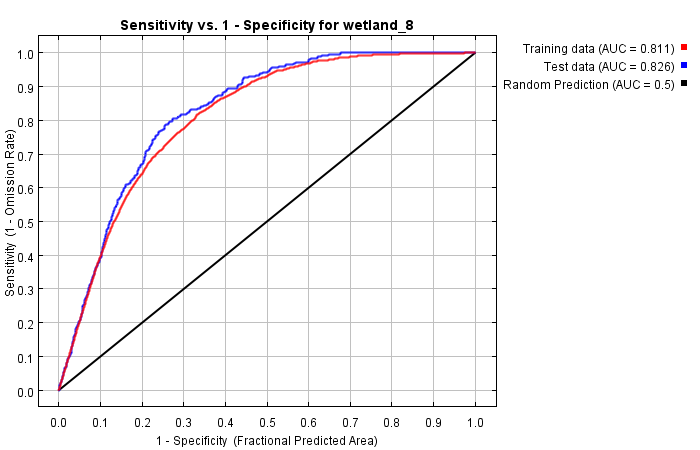

Supplement: Supplemental Information 2 [file peerj-12-18586-s002.zip › Maxent_data2/2.5mBIOssp245_2081-2100/RES/plots/wetland_8_roc.png]

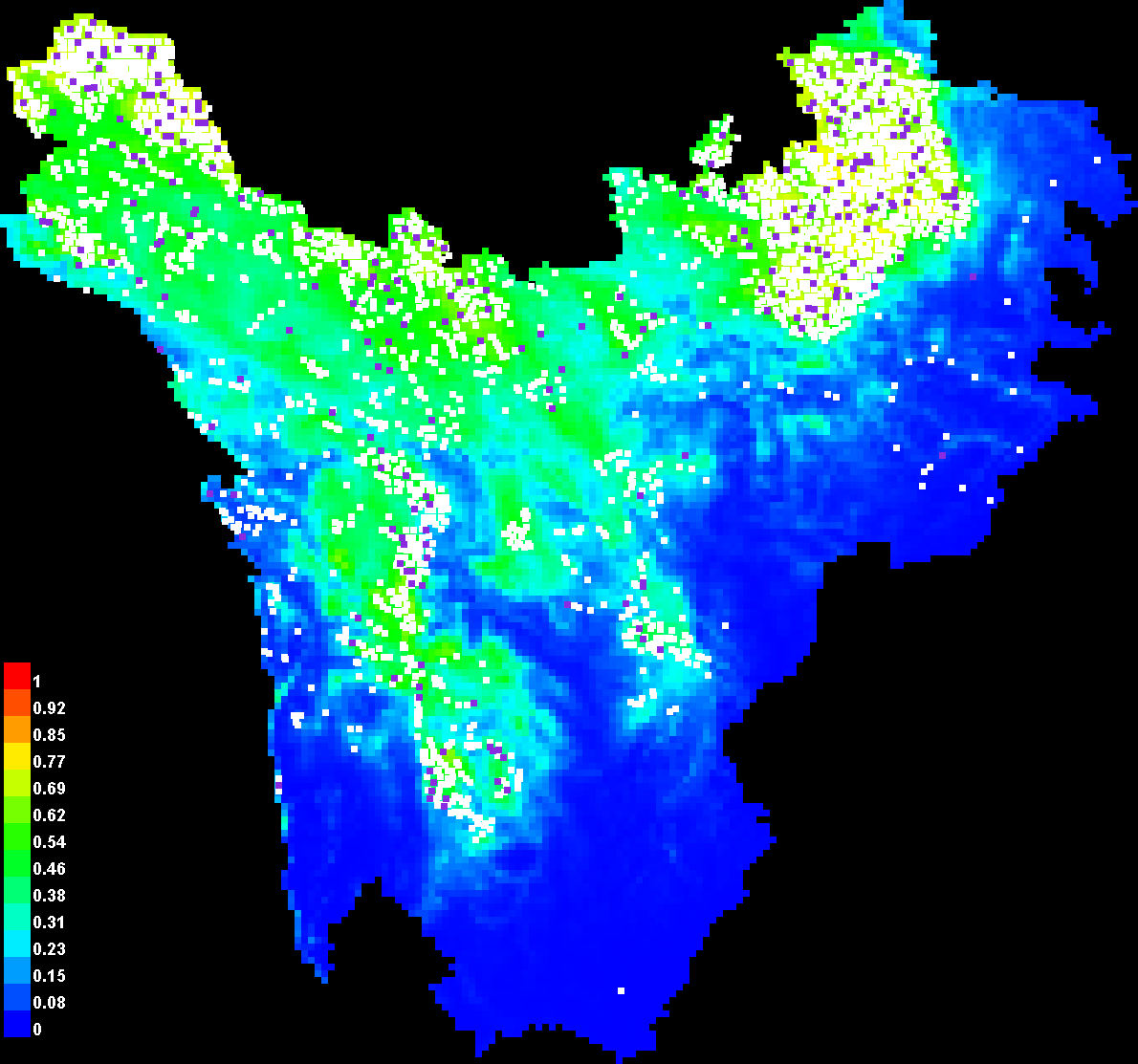

Supplement: Supplemental Information 2 [file peerj-12-18586-s002.zip › Maxent_data2/2.5mBIOssp245_2081-2100/RES/plots/wetland_9.png]

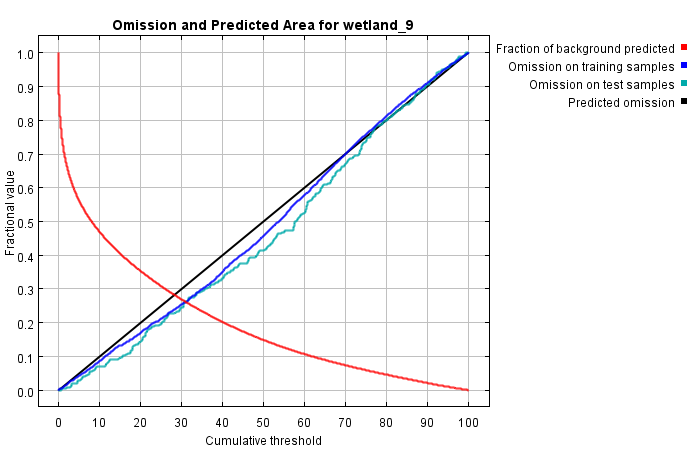

Supplement: Supplemental Information 2 [file peerj-12-18586-s002.zip › Maxent_data2/2.5mBIOssp245_2081-2100/RES/plots/wetland_9_omission.png]

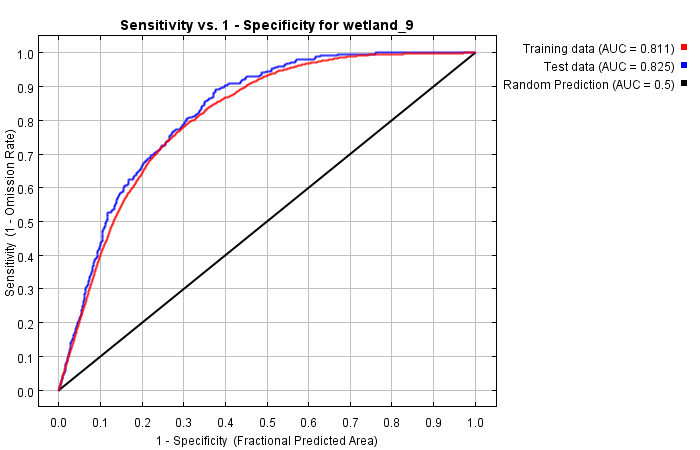

Supplement: Supplemental Information 2 [file peerj-12-18586-s002.zip › Maxent_data2/2.5mBIOssp245_2081-2100/RES/plots/wetland_9_roc.png]

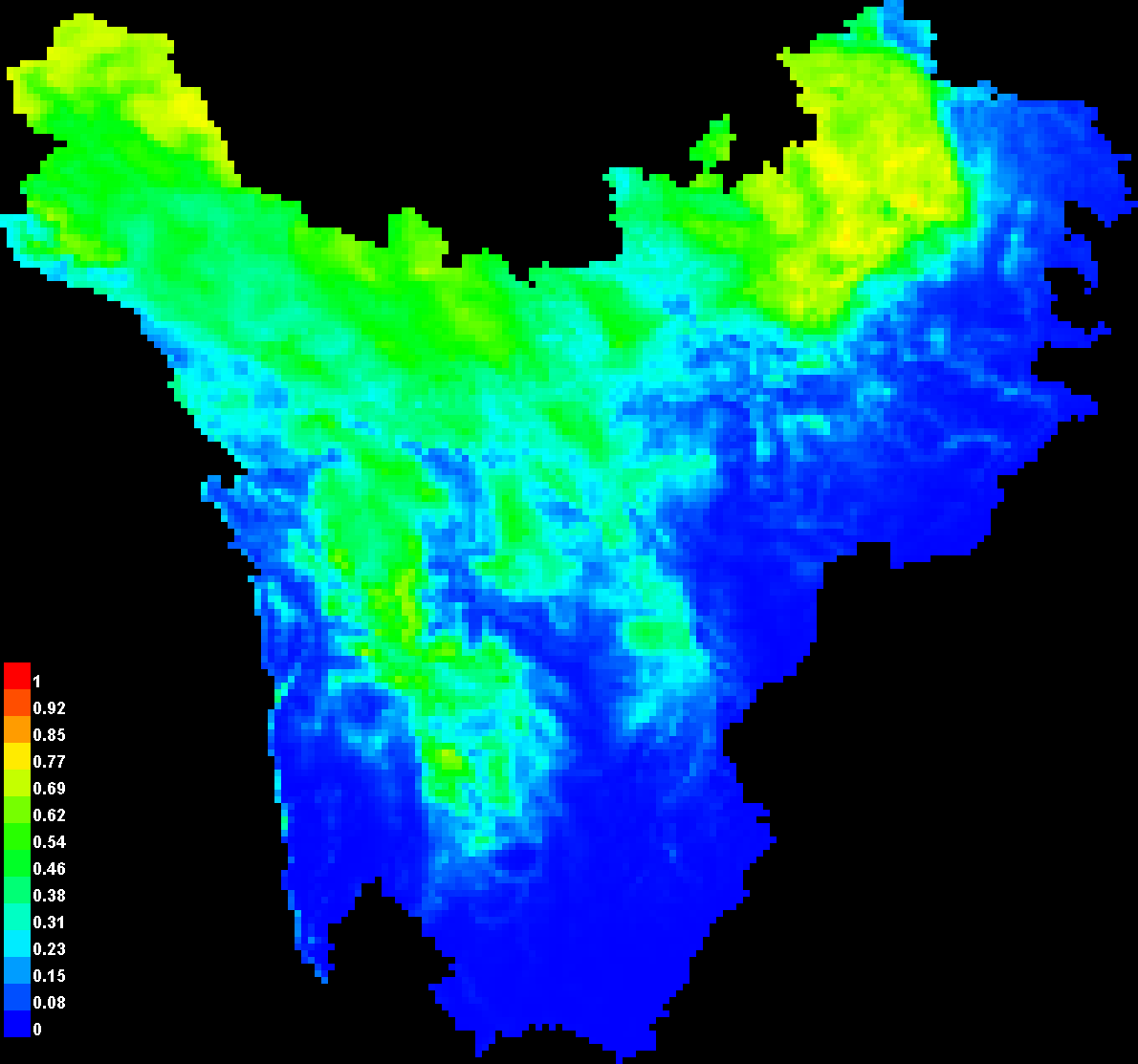

Supplement: Supplemental Information 2 [file peerj-12-18586-s002.zip › Maxent_data2/2.5mBIOssp245_2081-2100/RES/plots/wetland_avg.png]

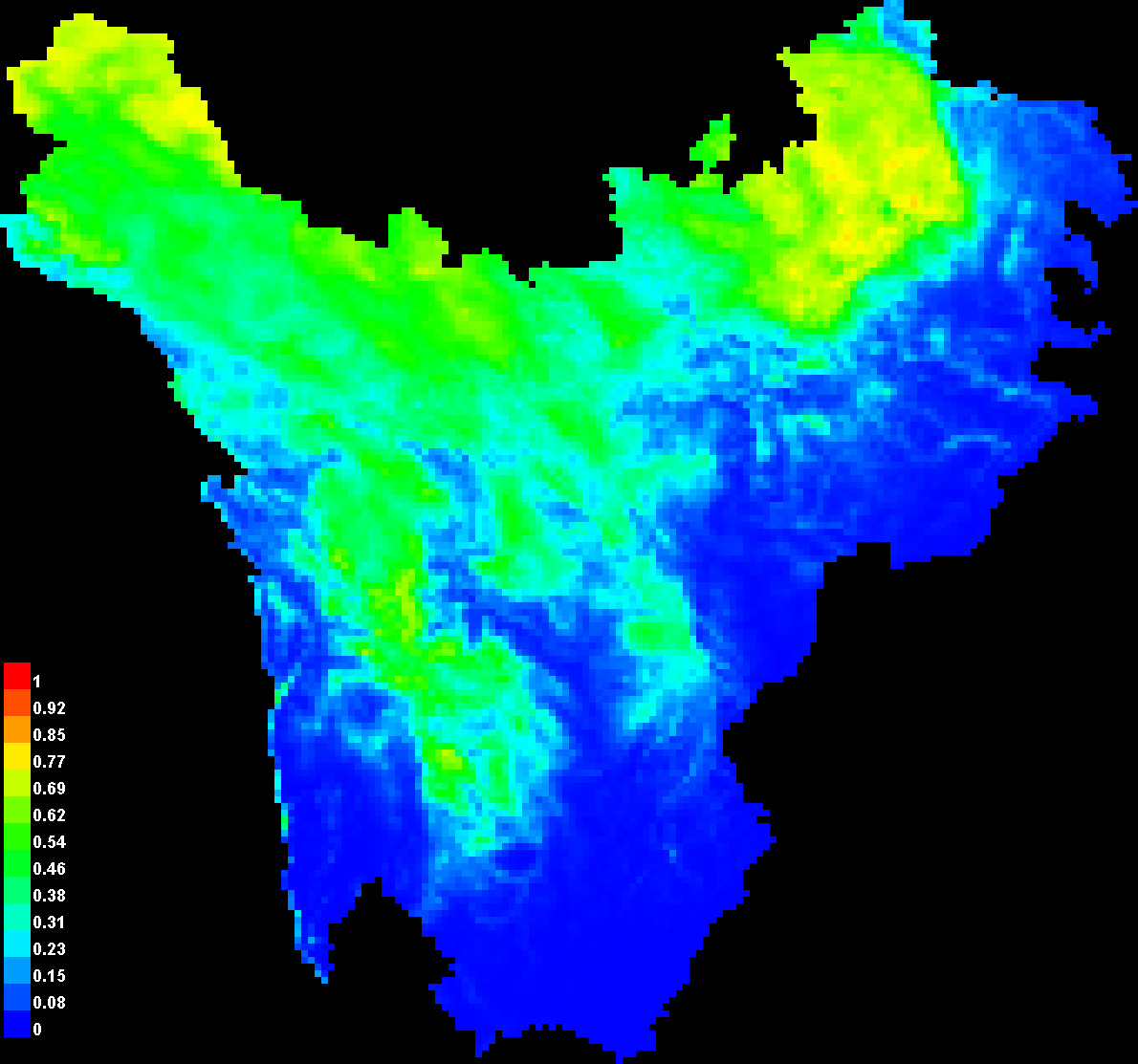

Supplement: Supplemental Information 2 [file peerj-12-18586-s002.zip › Maxent_data2/2.5mBIOssp245_2081-2100/RES/plots/wetland_max.png]

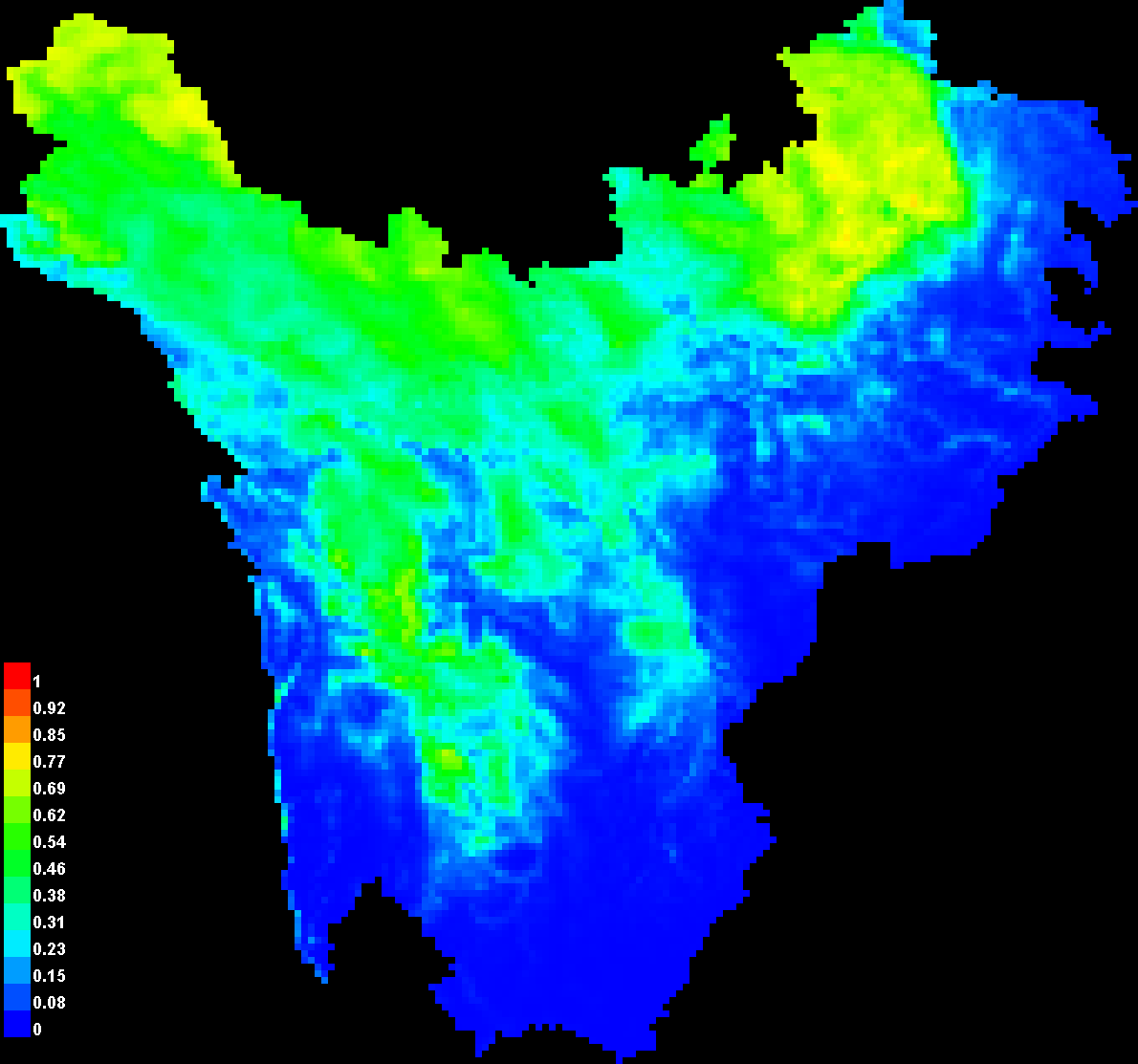

Supplement: Supplemental Information 2 [file peerj-12-18586-s002.zip › Maxent_data2/2.5mBIOssp245_2081-2100/RES/plots/wetland_median.png]

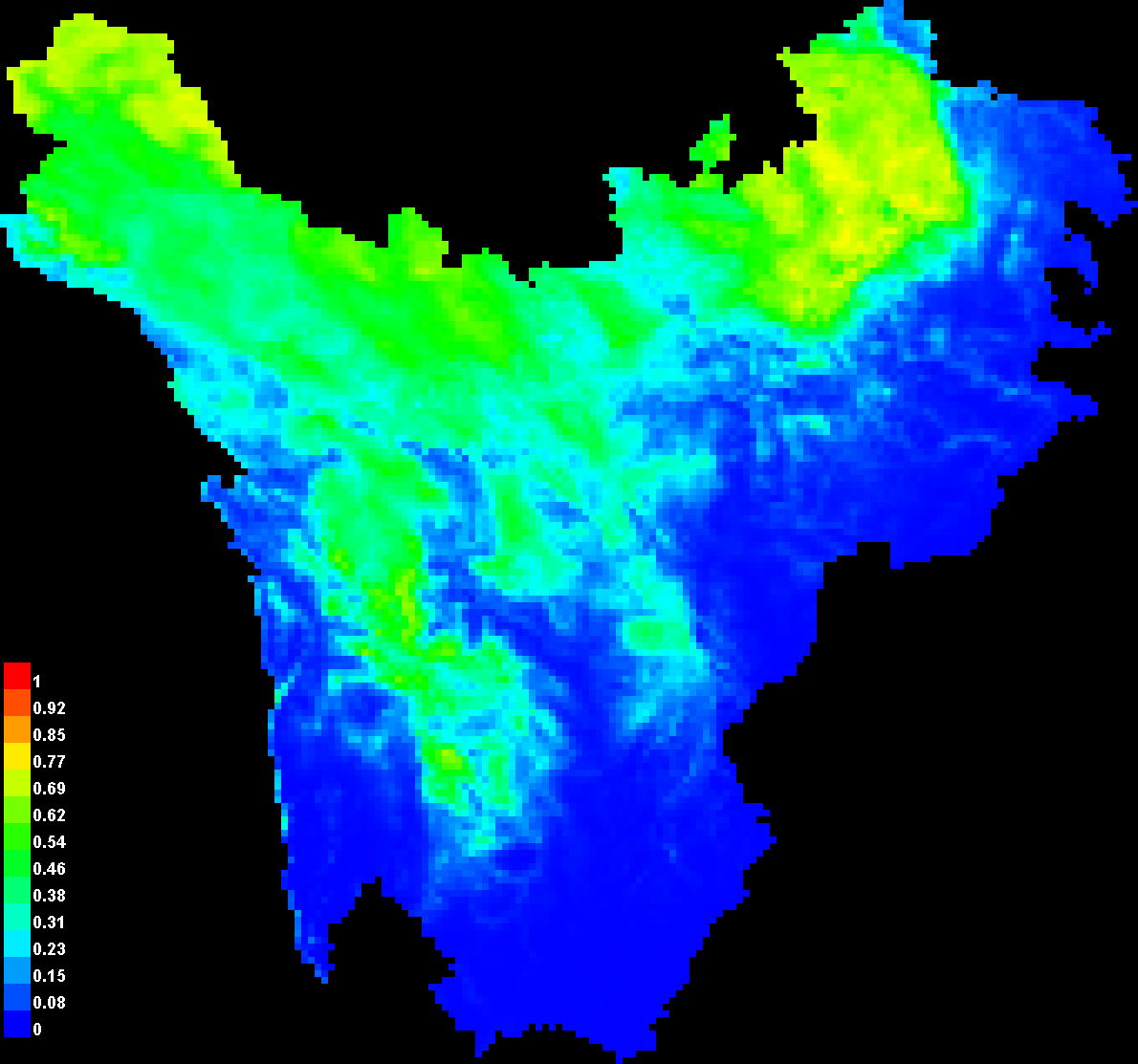

Supplement: Supplemental Information 2 [file peerj-12-18586-s002.zip › Maxent_data2/2.5mBIOssp245_2081-2100/RES/plots/wetland_min.png]

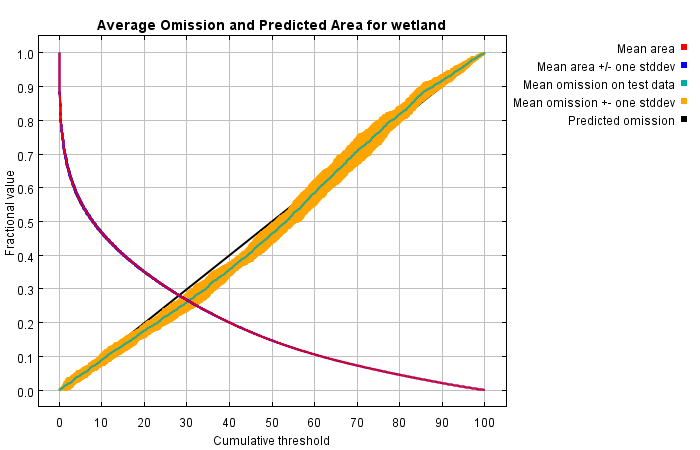

Supplement: Supplemental Information 2 [file peerj-12-18586-s002.zip › Maxent_data2/2.5mBIOssp245_2081-2100/RES/plots/wetland_omission.png]

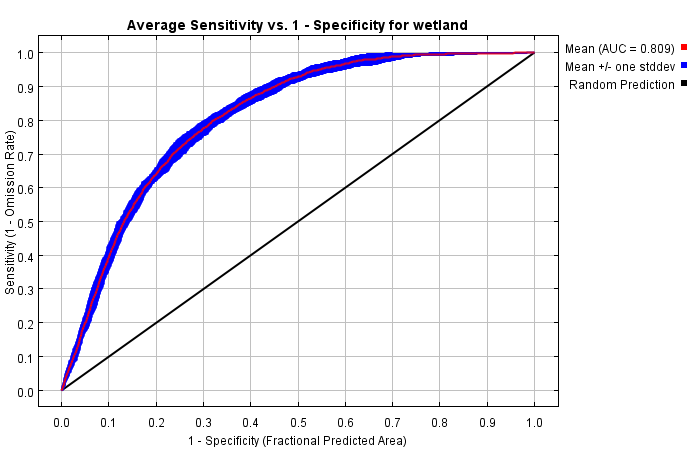

Supplement: Supplemental Information 2 [file peerj-12-18586-s002.zip › Maxent_data2/2.5mBIOssp245_2081-2100/RES/plots/wetland_roc.png]

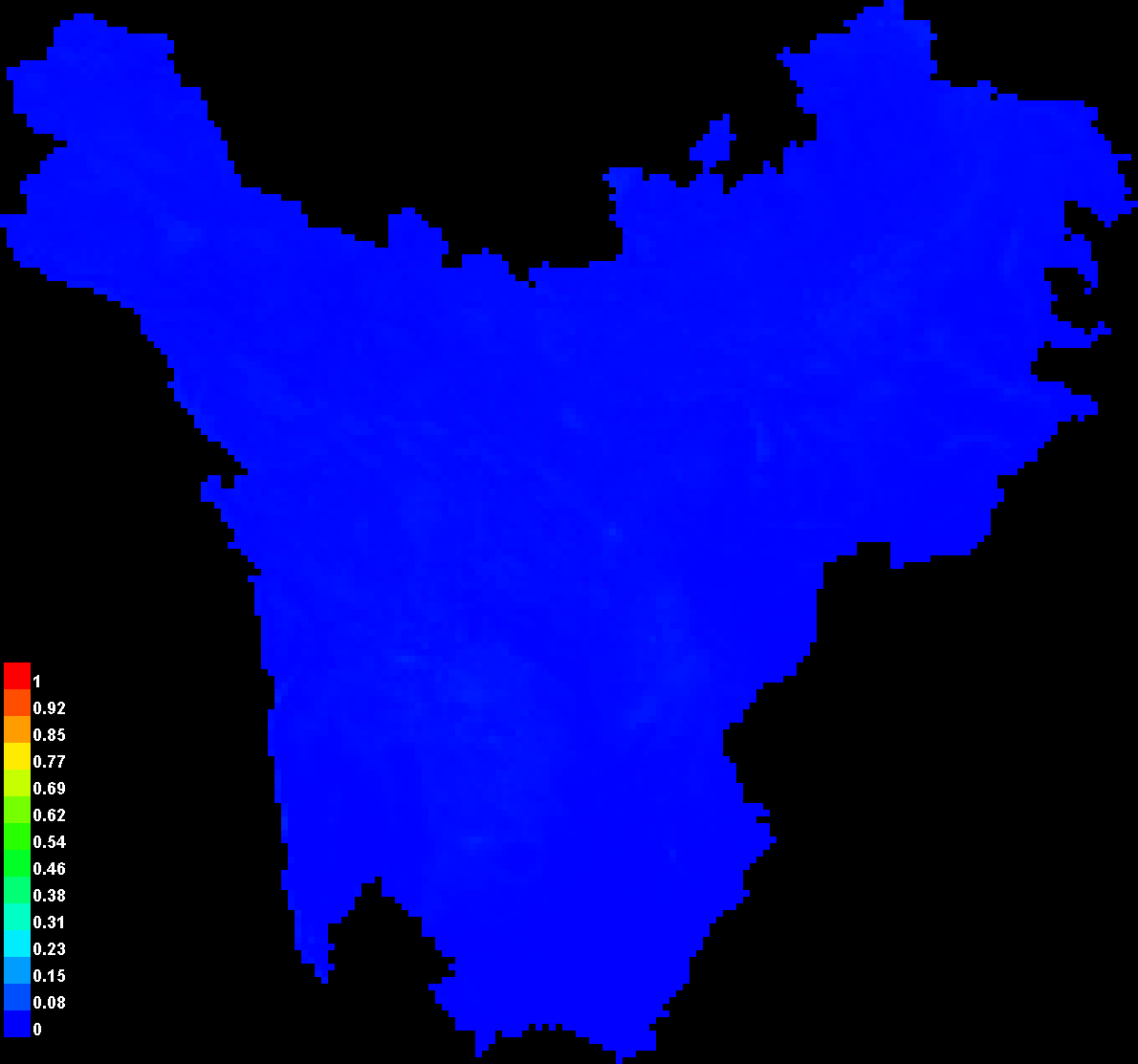

Supplement: Supplemental Information 2 [file peerj-12-18586-s002.zip › Maxent_data2/2.5mBIOssp245_2081-2100/RES/plots/wetland_stddev.png]

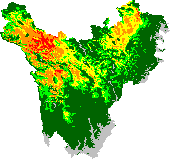

Supplement: Supplemental Information 3 [file peerj-12-18586-s003.zip › DIV-GIS_data/2.5mBIOssp126_2021-2040/25mbiossp126_2021-2040_bioclim.bmp]

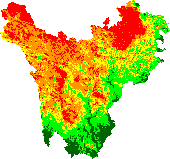

Supplement: Supplemental Information 3 [file peerj-12-18586-s003.zip › DIV-GIS_data/2.5mBIOssp126_2021-2040/25mbiossp126_2021-2040_domain.bmp]

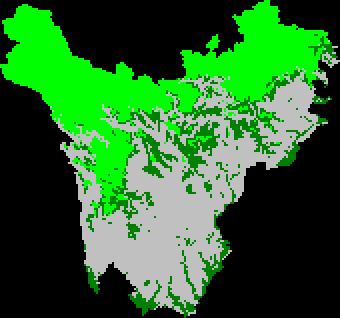

Supplement: Supplemental Information 4 [file peerj-12-18586-s004.zip › GARPP_data/2.5mBIOssp126_2021-2040/task_000001_0.bmp]

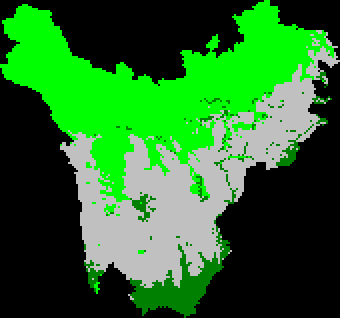

Supplement: Supplemental Information 4 [file peerj-12-18586-s004.zip › GARPP_data/2.5mBIOssp126_2021-2040/task_000002_0.bmp]

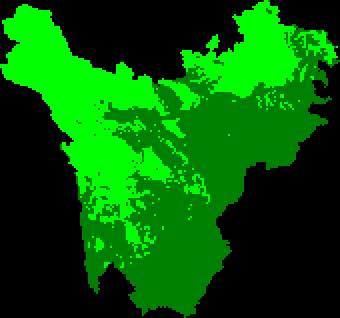

Supplement: Supplemental Information 4 [file peerj-12-18586-s004.zip › GARPP_data/2.5mBIOssp126_2021-2040/task_000003_0.bmp]

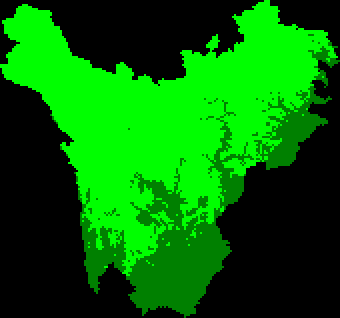

Supplement: Supplemental Information 4 [file peerj-12-18586-s004.zip › GARPP_data/2.5mBIOssp126_2021-2040/task_000004_0.bmp]

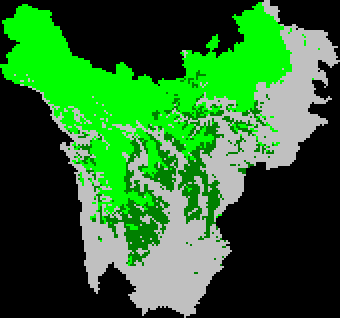

Supplement: Supplemental Information 4 [file peerj-12-18586-s004.zip › GARPP_data/2.5mBIOssp126_2021-2040/task_000005_0.bmp]

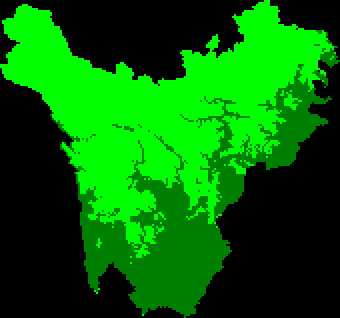

Supplement: Supplemental Information 4 [file peerj-12-18586-s004.zip › GARPP_data/2.5mBIOssp126_2021-2040/task_000006_0.bmp]

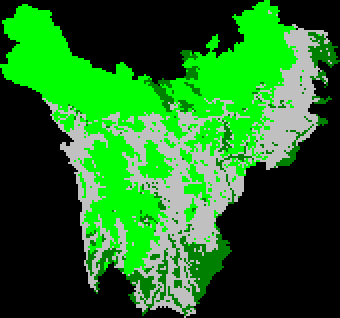

Supplement: Supplemental Information 4 [file peerj-12-18586-s004.zip › GARPP_data/2.5mBIOssp126_2021-2040/task_000007_0.bmp]

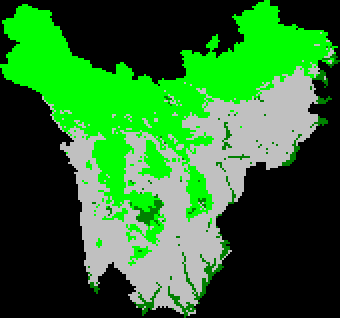

Supplement: Supplemental Information 4 [file peerj-12-18586-s004.zip › GARPP_data/2.5mBIOssp126_2021-2040/task_000008_0.bmp]

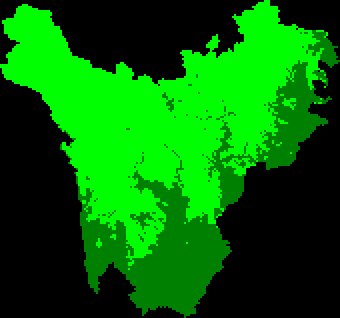

Supplement: Supplemental Information 4 [file peerj-12-18586-s004.zip › GARPP_data/2.5mBIOssp126_2021-2040/task_000009_0.bmp]

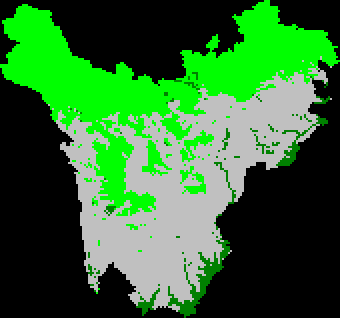

Supplement: Supplemental Information 4 [file peerj-12-18586-s004.zip › GARPP_data/2.5mBIOssp126_2021-2040/task_000010_0.bmp]

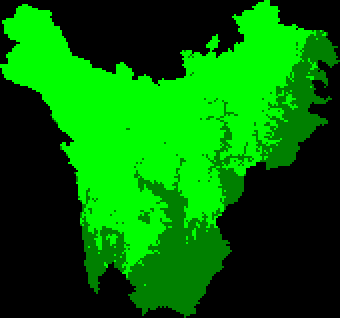

Supplement: Supplemental Information 4 [file peerj-12-18586-s004.zip › GARPP_data/2.5mBIOssp126_2021-2040/task_000011_0.bmp]

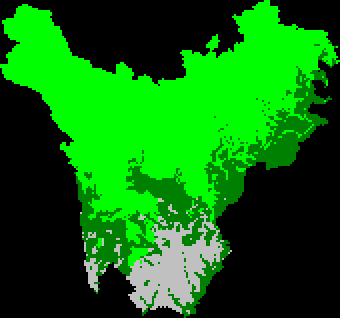

Supplement: Supplemental Information 4 [file peerj-12-18586-s004.zip › GARPP_data/2.5mBIOssp126_2021-2040/task_000012_0.bmp]

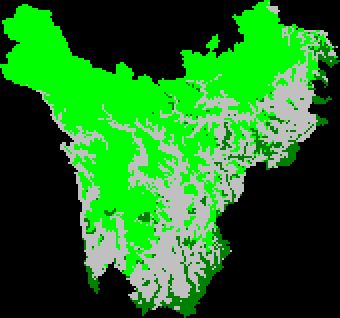

Supplement: Supplemental Information 4 [file peerj-12-18586-s004.zip › GARPP_data/2.5mBIOssp126_2021-2040/task_000013_0.bmp]

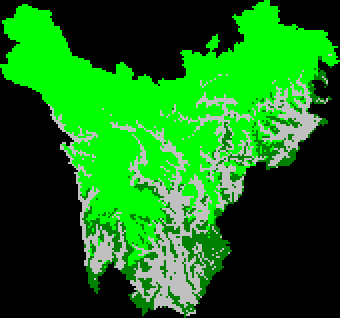

Supplement: Supplemental Information 4 [file peerj-12-18586-s004.zip › GARPP_data/2.5mBIOssp126_2021-2040/task_000014_0.bmp]

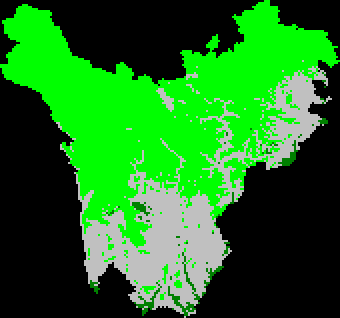

Supplement: Supplemental Information 4 [file peerj-12-18586-s004.zip › GARPP_data/2.5mBIOssp126_2021-2040/task_000015_0.bmp]

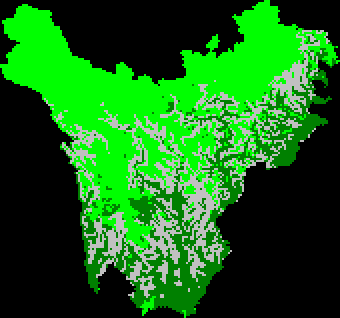

Supplement: Supplemental Information 4 [file peerj-12-18586-s004.zip › GARPP_data/2.5mBIOssp126_2021-2040/task_000016_0.bmp]

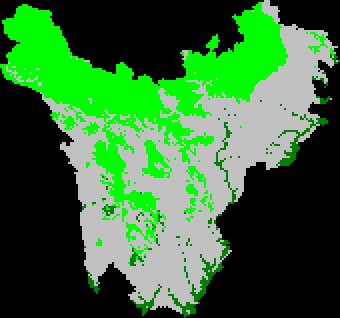

Supplement: Supplemental Information 4 [file peerj-12-18586-s004.zip › GARPP_data/2.5mBIOssp126_2021-2040/task_000017_0.bmp]

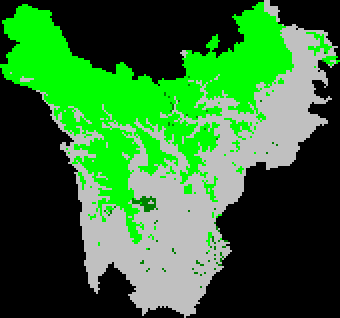

Supplement: Supplemental Information 4 [file peerj-12-18586-s004.zip › GARPP_data/2.5mBIOssp126_2021-2040/task_000018_0.bmp]

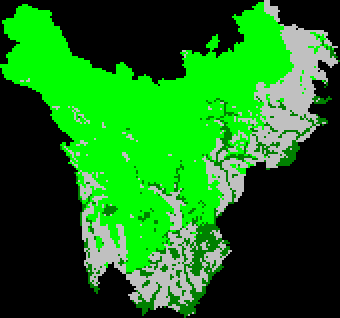

Supplement: Supplemental Information 4 [file peerj-12-18586-s004.zip › GARPP_data/2.5mBIOssp126_2021-2040/task_000019_0.bmp]

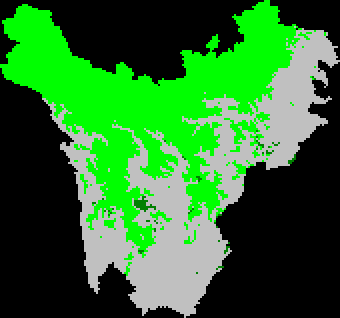

Supplement: Supplemental Information 4 [file peerj-12-18586-s004.zip › GARPP_data/2.5mBIOssp126_2021-2040/task_000020_0.bmp]
