# Supplementary material for: Spatiotemporal prediction of alpine wetlands under multi-climate scenarios in the west of Sichuan, China
Source: PeerJ. 2024 Nov 27;12:e18586. doi: 10.7717/peerj.18586 (PMC11608022; doi:10.7717/peerj.18586)
Supplement: Supplemental Information 1 [file peerj-12-18586-s001.zip › Maxent_data1/2.5mBIOssp126_2081-2100/RES/wetland_9.html]

Maxent model for wetland\_9


# Maxent model for wetland\_9

  
 This page contains some analysis of the Maxent model for wetland\_9, created Sat Oct 31 14:44:40 GMT+08:00 2020 using Maxent version 3.4.1. If you would like to do further analyses, the raw data used here is linked to at the end of this page.  
  

---

## Analysis of omission/commission

The following picture shows the omission rate and predicted area as a function of the cumulative threshold. The omission rate is is calculated both on the training presence records, and (if test data are used) on the test records. The omission rate should be close to the predicted omission, because of the definition of the cumulative threshold.
  
  
  
 The next picture is the receiver operating characteristic (ROC) curve for the same data. Note that the specificity is defined using predicted area, rather than true commission (see the paper by Phillips, Anderson and Schapire cited on the help page for discussion of what this means). This implies that the maximum achievable AUC is less than 1. If test data is drawn from the Maxent distribution itself, then the maximum possible test AUC would be 0.795 rather than 1; in practice the test AUC may exceed this bound.
  
  
  
  
Some common thresholds and corresponding omission rates are as follows. If test data are available, binomial probabilities are calculated exactly if the number of test samples is at most 25, otherwise using a normal approximation to the binomial. These are 1-sided p-values for the null hypothesis that test points are predicted no better than by a random prediction with the same fractional predicted area. The "Balance" threshold minimizes 6 \* training omission rate + .04 \* cumulative threshold + 1.6 \* fractional predicted area.  
  

|  |  |  |  |  |  |  |  |  |  |  |  |  |  |  |  |  |  |  |  |  |  |  |  |  |  |  |  |  |  |  |  |  |  |  |  |  |  |  |  |  |  |  |  |  |  |  |  |  |  |  |  |  |  |  |  |  |  |  |  |  |  |  |  |  |  |  |  |  |  |  |  |  |  |  |  |  |  |  |  |  |  |  |  |
| --- | --- | --- | --- | --- | --- | --- | --- | --- | --- | --- | --- | --- | --- | --- | --- | --- | --- | --- | --- | --- | --- | --- | --- | --- | --- | --- | --- | --- | --- | --- | --- | --- | --- | --- | --- | --- | --- | --- | --- | --- | --- | --- | --- | --- | --- | --- | --- | --- | --- | --- | --- | --- | --- | --- | --- | --- | --- | --- | --- | --- | --- | --- | --- | --- | --- | --- | --- | --- | --- | --- | --- | --- | --- | --- | --- | --- | --- | --- | --- | --- | --- | --- | --- |
| Cumulative threshold | Logistic threshold | Description | Fractional predicted area | Training omission rate | Test omission rate | P-value || 1.000 | 0.062 | Fixed cumulative value 1 | 0.730 | 0.007 | 0.008 | 1.725E-21 || 5.000 | 0.184 | Fixed cumulative value 5 | 0.570 | 0.040 | 0.020 | 1.877E-40 || 10.000 | 0.265 | Fixed cumulative value 10 | 0.477 | 0.084 | 0.059 | 1.923E-50 || 0.010 | 0.003 | Minimum training presence | 0.956 | 0.000 | 0.000 | 3.017E-4 || 11.735 | 0.287 | 10 percentile training presence | 0.452 | 0.100 | 0.090 | 1.948E-49 || 31.836 | 0.431 | Equal training sensitivity and specificity | 0.260 | 0.260 | 0.254 | 0E0 || 29.731 | 0.420 | Maximum training sensitivity plus specificity | 0.276 | 0.239 | 0.230 | 0E0 || 32.600 | 0.434 | Equal test sensitivity and specificity | 0.255 | 0.270 | 0.254 | 0E0 || 27.709 | 0.409 | Maximum test sensitivity plus specificity | 0.291 | 0.226 | 0.211 | 0E0 || 1.645 | 0.088 | Balance training omission, predicted area and threshold value | 0.686 | 0.011 | 0.012 | 1.132E-25 || 6.099 | 0.207 | Equate entropy of thresholded and original distributions | 0.545 | 0.049 | 0.043 | 3.075E-40 |

  
  

---

## Pictures of the model

This is a representation of the Maxent model for wetland\_9. Warmer colors show areas with better predicted conditions. White dots show the presence locations used for training, while violet dots show test locations. Click on the image for a full-size version.  
  
   
  
(A link to the Explain tool was not made for this model. The model uses product features, while the Explain tool can only be used for additive models.)  
  
  

---

## Analysis of variable contributions

  
The following table gives estimates of relative contributions of the environmental variables to the Maxent model. To determine the first estimate, in each iteration of the training algorithm, the increase in regularized gain is added to the contribution of the corresponding variable, or subtracted from it if the change to the absolute value of lambda is negative. For the second estimate, for each environmental variable in turn, the values of that variable on training presence and background data are randomly permuted. The model is reevaluated on the permuted data, and the resulting drop in training AUC is shown in the table, normalized to percentages. As with the variable jackknife, variable contributions should be interpreted with caution when the predictor variables are correlated.  
  

|  |  |  |  |  |  |  |  |  |  |  |  |  |  |  |  |  |  |  |  |  |  |  |  |  |  |  |  |  |  |  |  |  |  |  |  |  |  |  |  |  |  |  |  |  |  |  |  |  |  |  |  |  |  |  |  |  |  |  |  |  |  |  |  |  |  |
| --- | --- | --- | --- | --- | --- | --- | --- | --- | --- | --- | --- | --- | --- | --- | --- | --- | --- | --- | --- | --- | --- | --- | --- | --- | --- | --- | --- | --- | --- | --- | --- | --- | --- | --- | --- | --- | --- | --- | --- | --- | --- | --- | --- | --- | --- | --- | --- | --- | --- | --- | --- | --- | --- | --- | --- | --- | --- | --- | --- | --- | --- | --- | --- | --- | --- |
| Variable | Percent contribution | Permutation importance || 2.5mbiossp126\_2081-2100\_7 | 73.3 | 38.4 |
| 2.5mbiossp126\_2081-2100\_2 | 6.7 | 20.6 |
| 2.5mbiossp126\_2081-2100\_15 | 5.6 | 16.4 |
| 2.5mbiossp126\_2081-2100\_12 | 3.2 | 2.8 |
| 2.5mbiossp126\_2081-2100\_13 | 3 | 2.8 |
| 2.5mbiossp126\_2081-2100\_4 | 1.7 | 1 |
| 2.5mbiossp126\_2081-2100\_6 | 1.5 | 3.2 |
| 2.5mbiossp126\_2081-2100\_11 | 1 | 0 |
| 2.5mbiossp126\_2081-2100\_3 | 0.7 | 2.7 |
| wc2.1\_2.5m\_elev | 0.7 | 0.7 |
| 2.5mbiossp126\_2081-2100\_19 | 0.6 | 1.7 |
| 2.5mbiossp126\_2081-2100\_16 | 0.5 | 4 |
| 2.5mbiossp126\_2081-2100\_9 | 0.4 | 0 |
| 2.5mbiossp126\_2081-2100\_18 | 0.3 | 2.1 |
| 2.5mbiossp126\_2081-2100\_5 | 0.3 | 1.4 |
| 2.5mbiossp126\_2081-2100\_14 | 0.3 | 0 |
| 2.5mbiossp126\_2081-2100\_8 | 0.1 | 2 |
| 2.5mbiossp126\_2081-2100\_17 | 0 | 0.1 |
| 2.5mbiossp126\_2081-2100\_1 | 0 | 0 |
| wc2.1\_2.5m\_bioc\_bcc-csm2-mr\_ssp126\_2081-2100 | 0 | 0 |
| 2.5mbiossp126\_2081-2100\_10 | 0 | 0 |

  
  
  

---

## Raw data outputs and control parameters

  
The data used in the above analysis is contained in the next links. Please see the Help button for more information on these.  
The model applied to the training environmental layers  
The coefficients of the model  
The omission and predicted area for varying cumulative and raw thresholds  
The prediction strength at the training and (optionally) test presence sites  
Results for all species modeled in the same Maxent run, with summary statistics and (optionally) jackknife results  
  
  
Regularized training gain is 0.608, training AUC is 0.810, unregularized training gain is 0.664.  
Unregularized test gain is 0.731.  
Test AUC is 0.823, standard deviation is 0.010 (calculated as in DeLong, DeLong & Clarke-Pearson 1988, equation 2).  
Algorithm terminated after 500 iterations (11 seconds).  
  
The follow settings were used during the run:  
2310 presence records used for training, 256 for testing.  
10583 points used to determine the Maxent distribution (background points and presence points).  
Environmental layers used (all continuous): 2.5mbiossp126\_2081-2100\_1 2.5mbiossp126\_2081-2100\_10 2.5mbiossp126\_2081-2100\_11 2.5mbiossp126\_2081-2100\_12 2.5mbiossp126\_2081-2100\_13 2.5mbiossp126\_2081-2100\_14 2.5mbiossp126\_2081-2100\_15 2.5mbiossp126\_2081-2100\_16 2.5mbiossp126\_2081-2100\_17 2.5mbiossp126\_2081-2100\_18 2.5mbiossp126\_2081-2100\_19 2.5mbiossp126\_2081-2100\_2 2.5mbiossp126\_2081-2100\_3 2.5mbiossp126\_2081-2100\_4 2.5mbiossp126\_2081-2100\_5 2.5mbiossp126\_2081-2100\_6 2.5mbiossp126\_2081-2100\_7 2.5mbiossp126\_2081-2100\_8 2.5mbiossp126\_2081-2100\_9 wc2.1\_2.5m\_bioc\_bcc-csm2-mr\_ssp126\_2081-2100 wc2.1\_2.5m\_elev  
Regularization values: linear/quadratic/product: 0.050, categorical: 0.250, threshold: 1.000, hinge: 0.500  
Feature types used: hinge product linear quadratic  
outputformat: logistic  
outputdirectory: C:\Users\wangh\Desktop\Wetland Predict1028\SSP model\Maxent\2.5mBIOssp126\_2081-2100\RES  
samplesfile: C:\Users\wangh\Desktop\Wetland Predict1028\SSP model\WetlandPoint.csv  
environmentallayers: C:\Users\wangh\Desktop\Wetland Predict1028\SSP model\Maxent\2.5mBIOssp126\_2081-2100  
testsamplesfile: C:\Users\wangh\Desktop\Wetland Predict1028\SSP model\WetlandPoint.csv  
replicates: 10  
Command line used:
