# Supplementary material for: Spatiotemporal prediction of alpine wetlands under multi-climate scenarios in the west of Sichuan, China
Source: PeerJ. 2024 Nov 27;12:e18586. doi: 10.7717/peerj.18586 (PMC11608022; doi:10.7717/peerj.18586)
Supplement: Supplemental Information 1 [file peerj-12-18586-s001.zip › Maxent_data1/2.5mBIOssp245_2021-2040/RES/wetland.html]

Replicated maxent model for wetland


# Replicated maxent model for wetland

  
 This page summarizes the results of 10-fold cross-validation for wetland, created Sat Oct 31 14:48:02 GMT+08:00 2020 using Maxent version 3.4.1. The individual models are here: [0] [1] [2] [3] [4] [5] [6] [7] [8] [9]  
  

---

## Analysis of omission/commission

The following picture shows the test omission rate and predicted area as a function of the cumulative threshold, averaged over the replicate runs. The omission rate should be close to the predicted omission, because of the definition of the cumulative threshold.
  
  
  
 The next picture is the receiver operating characteristic (ROC) curve for the same data, again averaged over the replicate runs. Note that the specificity is defined using predicted area, rather than true commission (see the paper by Phillips, Anderson and Schapire cited on the help page for discussion of what this means). The average test AUC for the replicate runs is 0.806, and the standard deviation is 0.013.
  
  
  

---

## Pictures of the model

The following two pictures show the point-wise mean and standard deviation of the 10 output grids. Other available summary grids are min, max and median.  
  
 
 
  
  

---

## Analysis of variable contributions

  
The following table gives estimates of relative contributions of the environmental variables to the Maxent model. To determine the first estimate, in each iteration of the training algorithm, the increase in regularized gain is added to the contribution of the corresponding variable, or subtracted from it if the change to the absolute value of lambda is negative. For the second estimate, for each environmental variable in turn, the values of that variable on training presence and background data are randomly permuted. The model is reevaluated on the permuted data, and the resulting drop in training AUC is shown in the table, normalized to percentages. As with the variable jackknife, variable contributions should be interpreted with caution when the predictor variables are correlated. Values shown are averages over replicate runs.  
  

|  |  |  |  |  |  |  |  |  |  |  |  |  |  |  |  |  |  |  |  |  |  |  |  |  |  |  |  |  |  |  |  |  |  |  |  |  |  |  |  |  |  |  |  |  |  |  |  |  |  |  |  |  |  |  |  |  |  |  |  |  |  |  |  |  |  |
| --- | --- | --- | --- | --- | --- | --- | --- | --- | --- | --- | --- | --- | --- | --- | --- | --- | --- | --- | --- | --- | --- | --- | --- | --- | --- | --- | --- | --- | --- | --- | --- | --- | --- | --- | --- | --- | --- | --- | --- | --- | --- | --- | --- | --- | --- | --- | --- | --- | --- | --- | --- | --- | --- | --- | --- | --- | --- | --- | --- | --- | --- | --- | --- | --- | --- |
| Variable | Percent contribution | Permutation importance || 2.5mbiossp245\_2021-2040\_7 | 71.4 | 15.1 |
| 2.5mbiossp245\_2021-2040\_2 | 6 | 24.8 |
| 2.5mbiossp245\_2021-2040\_15 | 5.8 | 22.1 |
| 2.5mbiossp245\_2021-2040\_13 | 5.8 | 0.2 |
| 2.5mbiossp245\_2021-2040\_3 | 2.8 | 6.5 |
| 2.5mbiossp245\_2021-2040\_4 | 2.4 | 17.6 |
| 2.5mbiossp245\_2021-2040\_6 | 1.2 | 1.2 |
| 2.5mbiossp245\_2021-2040\_12 | 1.2 | 1.1 |
| 2.5mbiossp245\_2021-2040\_5 | 1.1 | 3.5 |
| wc2.1\_2.5m\_elev | 0.7 | 0.5 |
| 2.5mbiossp245\_2021-2040\_18 | 0.3 | 2.3 |
| 2.5mbiossp245\_2021-2040\_11 | 0.3 | 0 |
| 2.5mbiossp245\_2021-2040\_16 | 0.2 | 1.2 |
| 2.5mbiossp245\_2021-2040\_9 | 0.2 | 1.2 |
| 2.5mbiossp245\_2021-2040\_8 | 0.1 | 2 |
| 2.5mbiossp245\_2021-2040\_14 | 0.1 | 0.1 |
| 2.5mbiossp245\_2021-2040\_19 | 0.1 | 0.6 |
| 2.5mbiossp245\_2021-2040\_1 | 0 | 0 |
| 2.5mbiossp245\_2021-2040\_17 | 0 | 0 |
| 2.5mbiossp245\_2021-2040\_10 | 0 | 0 |
| wc2.1\_2.5m\_bioc\_bcc-csm2-mr\_ssp245\_2021-2040 | 0 | 0 |

  
  
  

---

  
Command line to repeat this species model: java density.MaxEnt nowarnings noprefixes -E "" -E wetland outputformat=logistic "outputdirectory=C:\Users\wangh\Desktop\Wetland Predict1028\SSP model\Maxent\2.5mBIOssp245\_2021-2040\RES" "samplesfile=C:\Users\wangh\Desktop\Wetland Predict1028\SSP model\WetlandPoint.csv" "environmentallayers=C:\Users\wangh\Desktop\Wetland Predict1028\SSP model\Maxent\2.5mBIOssp245\_2021-2040" "testsamplesfile=C:\Users\wangh\Desktop\Wetland Predict1028\SSP model\WetlandPoint.csv" replicates=10
